# Microenvironment‐Responsive Prodrug‐Induced Pyroptosis Boosts Cancer Immunotherapy

**DOI:** 10.1002/advs.202101840

**Published:** 2021-10-27

**Authors:** Yao Xiao, Tian Zhang, Xianbin Ma, Qi‐Chao Yang, Lei‐Lei Yang, Shao‐Chen Yang, Mengyun Liang, Zhigang Xu, Zhi‐Jun Sun

**Affiliations:** ^1^ The State Key Laboratory Breeding Base of Basic Science of Stomatology (Hubei‐MOST) & Key Laboratory of Oral Biomedicine Ministry of Education School & Hospital of Stomatology Wuhan University Wuhan 430079 China; ^2^ Key Laboratory of Luminescence Analysis and Molecular Sensing (Southwest University) Ministry of Education School of Materials and Energy & Chongqing Engineering Research Center for Micro–Nano Biomedical Materials and Devices Southwest University Chongqing 400715 China

**Keywords:** immunotherapy, paclitaxel, prodru gs, pyroptosis, tumor microenvironment

## Abstract

The absence of tumor antigens leads to a low response rate, which represents a major challenge in immune checkpoint blockade (ICB) therapy. Pyroptosis, which releases tumor antigens and damage‐associated molecular patterns (DAMPs) that induce antitumor immunity and boost ICB efficiency, potentially leads to injury when occurring in normal tissues. Therefore, a strategy and highly efficient agent to induce tumor‐specific pyroptosis but reduce pyroptosis in normal tissues is urgently required. Here, a smart tumor microenvironmental reactive oxygen species (ROS)/glutathione (GSH) dual‐responsive nano‐prodrug (denoted as MCPP) with high paclitaxel (PTX) and photosensitizer purpurin 18 (P18) loading is rationally designed. The ROS/GSH dual‐responsive system facilitates the nano‐prodrug response to high ROS/GSH in the tumor microenvironment and achieves optimal drug release in tumors. ROS generated by P18 after laser irradiation achieves controlled release and induces tumor cell pyroptosis with PTX by chemo‐photodynamic therapy. Pyroptotic tumor cells release DAMPs, thus initiating adaptive immunity, boosting ICB efficiency, achieving tumor regression, generating immunological memory, and preventing tumor recurrence. Mechanistically, chemo‐photodynamic therapy and control‐release PTX synergistically induce gasdermin E (GSDME)‐related pyroptosis. It is speculated that inspired chemo‐photodynamic therapy using the presented nano‐prodrug strategy can be a smart strategy to trigger pyroptosis and augment ICB efficiency.

## Graphical abstract

A smart tumor microenvironmental reactive oxygen species/glutathione dual‐responsive nano‐prodrug (denoted as MCPP) with high paclitaxel and photosensitizer purpurin 18 loading is designed. Chemo‐photodynamic therapy using the presented nano‐prodrug strategy can be a smart strategy to trigger pyroptosis and augment the efficiency of immune checkpoint blockade therapy.

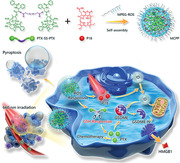

## 1 Introduction

Cancer immunotherapy has bloomed in the last several years owing to the great success of immune checkpoint blockade therapy (ICB) for multiple malignancies.^[^ ^1^ ^]^ However, the effective clinical use of anti‐PD‐1 agents is encumbered by their low response rate,^[^ ^2^ ^]^ which emphasizes the urgent need to improve the ICB response rate. Recent studies have demonstrated that treatment involving the promotion of tumor antigen release, initiation of antigen processing cells, and increased T‐cell infiltration may amplify the response rate of PD‐1 blockade therapy.^[^ ^3^ ^]^ However, agents that promote immunity can also impair normal tissues due to deficient tumor targeting.^[^ ^4^ ^]^ Thus, on the one hand, an agent proimmunity treatment should be explored to boost anti‐PD‐1 efficiency and, on the other hand, tumor specificity is required to avoid normal tissue damage.

Pyroptosis is a type of immunogenic cell death in which dying cells release antigens and robustly trigger antigen‐specific immune responses.^[^ ^5^ ^]^ Pyroptosis is executed by the gasdermin (GSDM) family.^[^ ^6^ ^]^ The linker of GSDM is specifically cleaved by certain caspases, generating a GSDM‐N fragment that can perforate membranes, thereby inducing pyroptosis and tumor cell pyrotosis, thus leading to antigen release and priming of the immune response.^[^ ^7^ ^]^ Pyroptosis‐induced chemotherapy or photodynamic therapy (PDT) can maximize the ICB response rate, and achieve appreciable control of tumor growth, resulting in robust and durable antitumor responses in cancer patients and in preclinical tumor models.^[^ ^3^, ^8^ ^]^ However, only certain cytotoxic drugs and photosensitizers are potent inducers of pyroptosis,^[^ ^5^ ^]^ and conventional cytotoxic agents and traditional photosensitizers without tumor targeting are inevitably distributed into normal tissues, leading to indiscriminate toxicity of both tumor tissue and normal tissues during treatment, possibly causing pyroptosis in normal tissues and triggering normal tissue damage.^[^ ^9^ ^]^ Therefore, a novel agent is urgently needed for robustly inducing pyroptosis with tumor microenvironment on‐target effects.

**Scheme 1:**
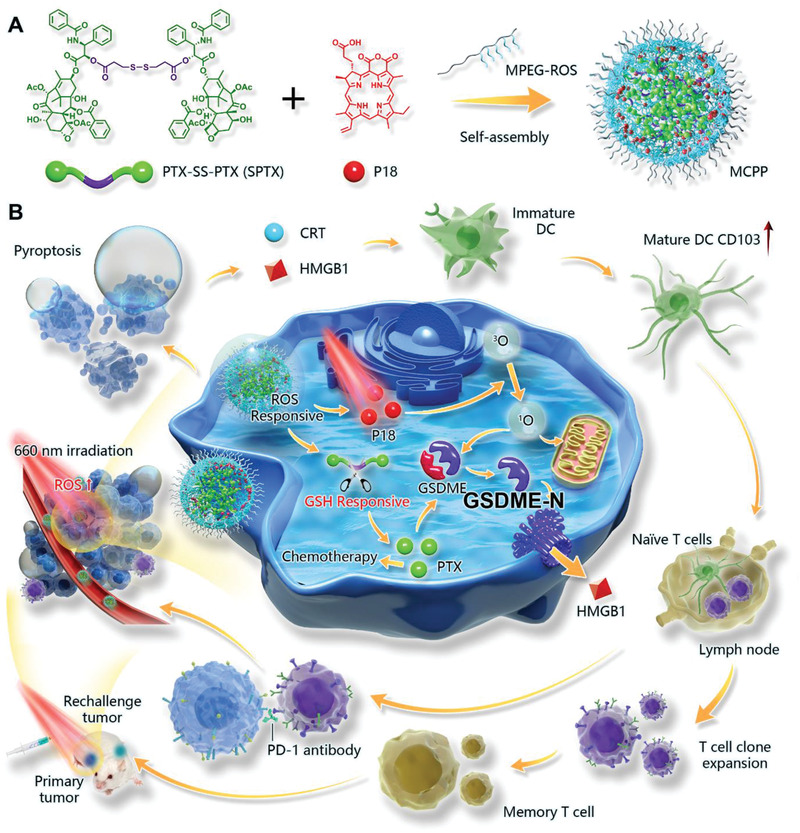
Schematic illustration of the MCPP for cancer immunotherapy by inducing pyroptosis. A) Synthesis of MCPP NPs. B) The mechanism of MCPP for boosting immune checkpoint therapy by inducing tumor pyroptosis, which is executed by GSDME.

Taking advantage of the higher reactive oxygen species (ROS)/glutathione (GSH) in the tumor microenvironment, a tumor microenvironment‐responsive nano‐prodrug was proposed for optimal drug release at tumor sites.^[^ ^10^ ^]^ Therefore, we attempted to simplify the preparation of an ROS‐responsive drug delivery system by using reversible addition–fragmentation chain transfer (RAFT) polymerization to polymerize a thioether functional monomer to achieve remotely controlled drug release. Meanwhile, for achieving synergistic pyroptosis‐inducing chemo‐photodynamic therapy, nanoparticles (NPs) were used to co‐encapsulate the GSH‐responsive PTX‐SS‐PTX (SPTX) dimer with a disulfate linker and purpurin 18 (P18) photosensitizer methoxypolyethylene glycols‐ 4‐cyano‐4‐(phenylcarbonothioylthio)pentanoic acid‐*b* *lock*‐P(M4)@SPTX/P18, MPEG‐CPPA‐*b*‐P(M4)@SPTX/P18, MCPP), which are regarded to be the inner core of nanoparticles.The GSH‐responsive dimeric drug strategy for SPTX not only effectively improved the paclitaxel (PTX) loading content, but also depleted GSH in the tumor environment,^[^ ^11^ ^]^ and amplified ROS cytotoxicity. The P18 photosensitizer enabled drug visualization. Moreover, ROS generated by P18 achieved controlled nano‐prodrug release and induced tumor cell pyroptosis. This versatile self‐assembled chemo‐photodynamic nanoparticle shorted as MCPP NPs and showed promise because of its high drug loading, controlled tumor microenvironmental drug release, deep tumor penetration, robust pyroptosis‐inducing ability, and a few systematic side effects. To evaluate the pyroptosis‐inducing ability of MCPP, we proposed an innovative pyroptosis index to quantify its dynamic pyroptosis‐inducing ability in vitro. After administering MCPP to tumor cells and followed by laser irradiation, MCPP induced rapid and durable GSDME‐dependent tumor cell pyroptosis. Pyroptosis cells release tumor antigens and damage‐associated molecular patterns (DAMPs), which promote dendritic cell (DC) maturation and initiate T‐cell clone expansion, prime the adaptive immune response, generate immunological memory, and enhance to cancer immune checkpoint blockade therapy, thus conferring tumor regression and long‐term survival (**Scheme** **1**).

## 2 Results and Discussion

### 2.1 Synthesis and Characterization of MCPP Nanoparticles

**Figure 1:**
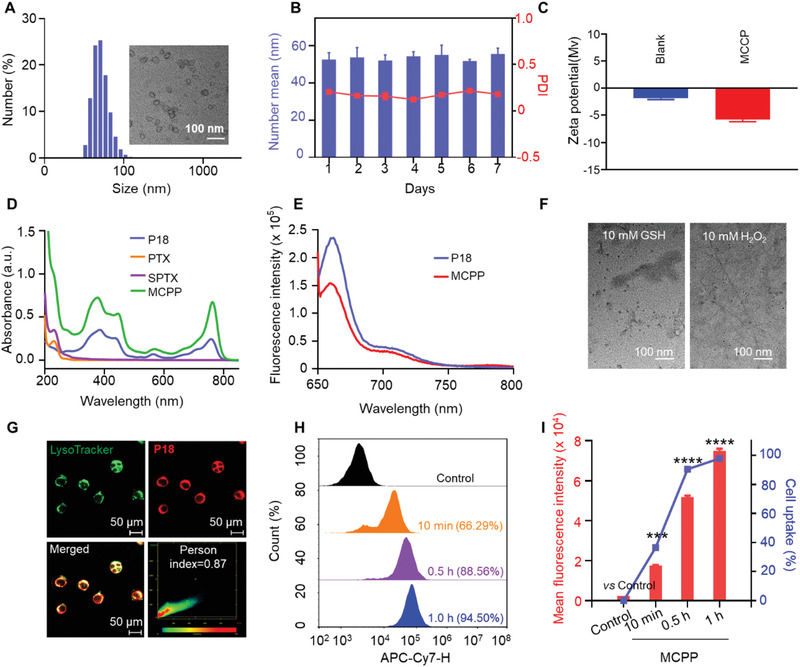
Characterization and cellular uptake behaviors of MCPP nanoparticles. A) DLS profile and TEM image of MCPP NPs. B) The micellar stability of MCPP for 7 days (*n* = 3). C) Zeta potential of MPEG‐CPPA‐*b*‐P (M4) (Blank) and MCPP in water (*n* = 3). D) UV–vis absorption spectra of different formulations. E) Fluorescence emission spectra of free P18 and MCPP in DMF. F) TEM images of MCPP micelles after GSH or H_2_O_2_ treatment. G) Colocalization of MCPP with lysosomes in CT26 cells; CT26 cells were incubated with MCPP for 6 h, then treated with LysoTracker (green fluorescence) for 15 min and observed by CLSM. H) Flow cytometry analysis of the MCPP signal in CT26 cells at various time points. I) Quantitative analysis of the flow cytometry results revealed that the cellular uptake of MCPP occurred in a time‐dependent manner. (*p < 0.05, **p < 0.01, ***p < 0.001, and ****p < 0.0001).

To achieve tumor microenvironment‐responsive release, the synthetic route for monomer 4 (M4) with a thioether moiety linker that responds to ROS is shown in Figure S1 (Supporting Information). The MPEG‐CPPA‐*b*‐P (M4) copolymer was synthesized by RAFT polymerization of the copolymerizing M4 (Figure S1, Supporting Information). The representative ^1^H NMR spectra of the copolymer of MPEG‐CPPA‐*b*‐P(M4) and its derivatives were determined and are shown in Figures S3–S7 (Supporting Information). The synthetic route for the SPTX dimeric drug with a GSH‐responsive disulfate linker is shown in Figure S2 (Supporting Information), and its chemical structure was confirmed by ^1^H NMR, ^13^C NMR, and mass spectrometry (Figures S8–S10, Supporting Information). Furthermore, the RAFT polymerization process was monitored by gel permeation chromatography (GPC). The molecular weights and PDI before or after polymerization were 16 800 and 1.21 or 25 400 and 1.49, respectively, suggesting a high level of control of the polymerization (Figure S11, Supporting Information).The MCPP NPs were prepared by co‐encapsulating SPTX and P18, based on hydrophilic and hydrophobic interactions. As shown in **Figure** **1**A, the MCPP NPs were homogeneous spheres, as observed by transmission electron microscopy (TEM). The hydrodynamic diameters and the surface *ζ* potential of the MCPP NPs were ≈52.69 ± 6.45 nm (polydispersity index, PDI = 0.20 ± 0.01) and −5.74 ± 0.76 mV, respectively, as measured by dynamic light scattering (DLS) (Figure 1C). Moreover, the DLS results in water, RPMI 1640, and 10% fetal bovine serum (FBS) showed little change over 7 days, indicating excellent stability of MCPP micelles (Figure 1B; Figure S12, Supporting Information). The UV–vis spectra of different samples are shown in Figure 1D. Strong absorbance was observed at 230 and 760 nm, indicating the efficient co‐encapsulation of PTX and P18. Moreover, the drug‐loading contents (LC%) of PTX and P18 were calculated as ≈17.6 and 1.3 wt% for MCPP, respectively, by the standard curve of UV–vis absorption against PTX and P18. In addition, the emissive fluorescence peak located at ≈662 nm of the MCPP NPs corresponded to free P18 (Figure 1E). These results suggest that the drugs were successfully encapsulated in the MCPP NPs. Because of the thioether linker of the MCPP copolymer and disulfide linker of SPTX, the H_2_O_2_/GSH dual‐responsive mechanism for sustained drug release of the MCPP NPs was confirmed using TEM imaging and drug release study. As shown in Figure 1F and Figure S13 (Supporting Information), small particles (<10 nm) were observed after treatment with 10 × 10^−3^ m GSH and 100 × 10^−3^ m H_2_O_2_. The subsequent drug release behavior of MCPP NPs showed that the loaded P18 was released quickly in the presence of H_2_O_2_, and the accumulative P18 release reached nearly 80% at 12 h. These results demonstrated that the MCPP NPs could be broken down at high GSH/ROS levels, leading to payload release.

### 2.2 Intracellular Trafficking

Cellular uptake and endocytic pathways of nanomaterials can critically affect the delivery efficiency and bioavailability of nanocarrier. In this study, mouse colon cancer CT26 cells were cocultured with MCPP for 0.5, 1, 4, 6, 8, and 12 h, and the CT26 cells were stained with LysoTracker, a bioprobe for lysosomes with green fluorescence emission. The MCPP signal (red) and lysosome signal (green) were detected by confocal laser scanning microscopy (CLSM, Zeiss 800) at the indicated time points. Changes in MCPP and lysosome signal location were observed, showing the entrance of nanoparticles into cells via an endocytosis pathway and the escape of the nano‐prodrugs from lysosome (Figure 1G; Figure S15, Supporting Information). The cellular uptake efficiency of MCPP by CT26 cells was assessed using flow cytometry and CLSM at the indicated time points. We found that intracellular MCPP reached a high level within 1 h and that the uptake of MCPP occurred in a time‐dependent manner (Figure 1H,I; Figure S16, Supporting Information). For optimal therapeutic PDT efficacy, nanoparticles require specific subcellular colocalization,^[^ ^12^ ^]^ and traditional cytoplasm‐localized PDT (CP‐PDT) has been reported to be less immunogenic than necrosis owing to the slow release of DAMPs during apoptosis.^[^ ^3^, ^13^ ^]^ Mitochondrial stress^[^ ^14^ ^]^ has been proposed to efficiently induce pyroptosis.^[^ ^15^ ^]^ Therefore, we evaluated the subcellular colocalization of MCPP with mitochondria. CT26 cells were incubated with MCPP, and MitoTracker, a bioprobe of mitochondria with green fluorescence, was used to mark the mitochondria. The MCPP and mitochondrial signals were detected using CLSM at different time points. We found that MCPP colocalized with mitochondria, and the MCPP signal was increased in a time‐dependent manner (Figure S17, Supporting Information), which demonstrated that the MCPP NPs were able to target mitochondria. These results indicate the excellent uptake efficiency and subcellular targeting ability of MCPP. Therefore, we speculated that MCPP might exhibit excellent PDT efficacy.

### 2.3 Cytotoxicity and Pyroptosis Evaluation In Vitro

**Figure 2:**
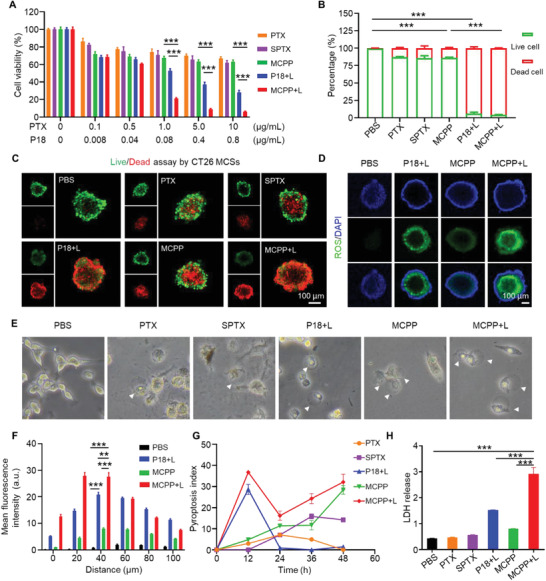
Cytotoxicity and antitumor immunity in vitro. A) Cell viability of CT26 cells treated with different concentrations of PTX, SPTX, P18+L, MCPP, MCPP+L, and CT26 incubated with each drug for 48 h; the cytotoxicity of each drug was dose dependent (*n* = 3). B) Quantitative analysis of live/dead assay by CT26 cells after treated with PBS, PTX, SPTX, P18+L, MCPP, and MCPP+L at a PTX dose of 30 µg mL^−1^ or a P18 dose of 2.4 µg mL^−1^ for 48 h (*n* = 3). C) CLSM image of the live (FDA, green signal)/dead (PI, red signal) assay staining for CT26 MCSs after the indicated treatment, the green and red signals represented live and dead cells, respectively. Scale bar = 100 µm. D) ROS detection in CT26 MCSs after treatment with PBS, P18+L, MCPP, and MCPP+L at a P18 dose of 5 µg mL^−1^ for 6 h; ROS were detected by DCFH‐DA (green signal). Scale bar = 100 µm. E) Representative bright‐field microscopy image of CT26 cells after treatment; the arrows indicated pyroptotic cells. F) Quantitative analysis of ROS in MCSs (*n* = 3). G) Pyroptosis index of CT26 cells after treatment (*n* = 5). H) Quantitative analysis of LDH release from CT26 cells after treatment (*n* = 3). (*p < 0.05, **p < 0.01, ***p < 0.001, and ****p < 0.0001).

Cytotoxicity and pyroptosis‐inducing efficiency are crucial for ensuring a successful antitumor ability. Therefore, we investigated the cytotoxicity of PTX, SPTX, free P18, and MCPP against CT26 cells, which is a common experimental model in pyroptosis research based on the rather high expression of GSDME.^[^ ^16^ ^]^ CT26 cells were incubated with serial dilutions of PTX, SPTX, free P18, and MCPP for 48 h, and cell viability and cell death were evaluated by 3‐(4,5‐dimethylthiazol‐2‐yl)‐2,5‐diphenyltetrazolium bromide (MTT) and live/dead assays, respectively. As shown in **Figure** **2**A, the cytotoxicity of PTX, SPTX, and MCPP exhibited a concentration‐dependent effect, and the cytotoxicity of MCPP (MCPP+laser, MCPP+L) and P18 (P18+laser, P18+L) was significantly enhanced after laser irradiation. The results of the live/dead assay also proved the enhanced cytotoxicity of MCPP (MCPP+L) and P18 (P18+L) after laser irradiation (Figure 2B; Figure S18, Supporting Information). Notably, the results of the live/dead assay also indicated that MCPP, after laser irradiation, was more toxic than PTX or SPTX. Consequently, we constructed multicellular spheroids (MCSs) of CT26 cells to imitate tumor tissues to explore cell death in tumor tissues.^[^ ^17^ ^]^ MCSs were incubated with phosphate‐buffered saline (PBS), PTX, SPTX, P18, and MCPP. A live/dead assay was performed to stain live or dead MCS cells. Consistent with the above results, we found that MCPP+L therapy was more toxic than PTX, SPTX, P18+L, and MCPP (Figure 2C; Figure S19, Supporting Information).

PDT is based on the principle of generating cytotoxic ROS to induce cell pyroptosis and tissue destruction under light activation by photosensitizers.^[^ ^7^, ^10^ ^]^ The 1,3 diphenylisobenzofuran (DPBF) probe was used to detect ROS production in MCPP micelles. UV–vis spectra showed that the absorption intensity of the characteristic peak decreased significantly, indicating greater ROS generation under 660 nm laser irradiation (Figure S14, Supporting Information). To confirm the pyroptosis‐inducing efficiency of P18+L and MCPP+L therapy, we first detected ROS triggered by P18+L and MCPP+L therapy. P18 and MCPP were incubated with CT26 cells, and a fluorescence probe (2′,7′‐dichlorofluorescin diacetate, DCFH–DA) was used to detect ROS. After irradiation, we found that the level of ROS, triggered by MCPP+L, was significantly higher than that triggered by P18+L or MCPP alone (Figures S20 and S21, Supporting Information). Moreover, MCSs were applied to simulate tumor tissue, and we also found that the level of ROS in MCSs, triggered by MCPP+L, was significantly higher than that triggered by P18+L or MCPP alone (Figure 2D,F; Figure S22, Supporting Information). This result indicated that MCPP+L could trigger more ROS in tumor tissue, paving the way for subsequent pyroptosis detection.

Tumor pyroptosis is executed by the GSDM family,^[^ ^7^ ^]^ which bears an N‐terminal pore‐forming domain and a C‐terminal inhibitory domain^[^ ^18^ ^]^ that are inactive until cleaved and activated by certain proteases, N‐terminal fragments of GSDMs form pores in the plasma membrane, leading to water influx, cell swelling and ballooning, and ultimately plasma membrane rupture.^[^ ^19^ ^]^ Therefore, we observed changes in cell morphology after different treatments at different time points, and the balloon‐like cells were considered pyroptotic cells.^[^ ^8^, ^20^ ^]^ Considering that the time of tumor cell pyroptosis was different under different treatments, we proposed an innovative pyroptosis index to better evaluate the ability of each treatment to induce pyroptosis. The ratio of the pyroptotic cell (balloon‐like cell) number to the total cell number in a snapshot multiplied by 100 was applied as the pyroptosis index of each treatment at the indicated time point. Over a 48 h continuous observation period, we observed a significant increase within 12 h, and the pyroptosis index reached its peak at 12 h in the P18+L and MCPP+L groups, ≈36.8 and ≈28.6 respectively, and pyroptosis index decreased from 12 to 24 h, and pyroptotic cells finally disappeared after 24 h in the P18+L group. However, in the MCPP+L group, the pyroptosis index remained elevated after 24 h and increased to 32.1 at 48 h. Different from the MCPP+L and P18+L groups, the increase in the pyroptosis index was initially slow during the first 24 h in the PTX, SPTX, and MCPP groups. The pyroptosis index of PTX reached its peak at 24 h, which was 7.09, and after 24 h, it decreased; pyroptotic cells finally disappeared at 48 h. The pyroptosis index of the SPTX group continued to increase after 24 h, peaking at 36 h, at 15.8. In the MCPP group, the pyroptosis index continued to rise after 36 h, increased to 28.4 at 48 h. (Figure 2E,G). Moreover, after MCPP+L therapy, CT26 tumor cells released high levels of lactate dehydrogenase (LDH) (Figure 2H). The change in the pyroptosis index indicated that MCPP+L treatment could integrate the therapeutic characteristics of PTX and P18, and exhibited a fast and persistent pyroptosis induction ability. The pyroptosis index is a promising method for evaluating and quantifying the dynamic proptosis‐inducing ability, but there is still much room for improvement in evaluating this index.

Pyroptotic cells release DAMPs, such as the translocation of calreticulin (CRT) to the surface of dying cells after endoplasmic reticulum (ER) stress and extracellular release of high mobility group box 1 (HMGB1).^[^ ^21^ ^]^ CLSM was then used to detect the subcellular location of CRT and HMGB1. We found that CRT was translocated on the cell surface, and that HMGB1 was released from the nucleus after MCPP+L treatment (Figure S23, Supporting Information).

### 2.4 Body Distribution of MCPP

**Figure 3:**
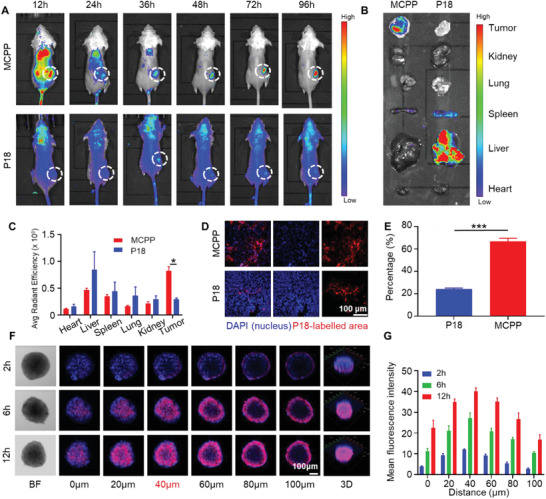
Body distribution and deep penetration of MCPP. A) In vivo image of MCPP and P18 fluorescence in CT26 tumor‐bearing mice body at 12, 24, 36, 48, 72, and 96 h after intravenous administration. B) Ex vivo fluorescence of MCPP and P18 in the heart, liver, spleen, lung, kidney, and tumor at 96 h after intravenous administration. C) Quantitative analysis of the fluorescence intensity of MCPP and P18 in the heart, liver, spleen, lung, kidney, and tumor 96 h after intravenous administration (*n* = 3). D) Fluorescence of MCPP and P18 in tumor tissue sections. Scale bar = 100 µm. E) Quantitative analysis of the fluorescence intensity of MCPP and P18 in tumor tissue sections (*n* = 3). F) CLSM images of MCPP penetration in CT26 MCSs. Bright field (BF), scale bar = 100 µm. G) Quantitative analysis of the fluorescence intensity of MCPP in CT26 MCSs at different *Z*‐axis (*n* = 3). (*p < 0.05, **p < 0.01, ***p < 0.001, and ****p < 0.0001).

Tumor‐targeting ability is one of the most vital prerequisites for antitumor efficiency in vivo,^[^ ^22^ ^]^ and it is important for inducing tumor‐specific pyroptosis. Drugs that specifically target tumors induce tumor cell pyroptosis and boost antitumor immunity.^[^ ^23^ ^]^ Tumor‐specific ability can avoid drug‐evoked normal tissue damage.^[^ ^24^ ^]^ Therefore, we investigated the body distribution and tumor accumulation of MCPP before in vivo antitumor experiments. Encouraged by the higher concentrations of ROS and GSH in tumor cells, our dual‐responsive MCPP system might be superior in improving accumulation at the tumor site.^[^ ^25^ ^]^ To investigate this assumption, MCPP and P18 were injected into CT26 tumor‐bearing BALB/c mice via the tail vein. Fluorescence live imaging was used to monitor the body distribution and tumor accumulation of MCPP and P18 fluorescence. 12 h after injection, MCPP began to accumulate at the tumor site. 36 h after injection, MCPP signal existed only at the tumor site. The MCPP signal at the tumor site continued 96 h post injection. In contrast to MCPP, P18 had no ability to target tumors; during the 96 h after injection, P18 did not significantly accumulate at tumor site (**Figure** **3**A). At 96 h post injection, the mice were euthanized, and the major organs were used for ex vivo imaging after surgical removal. We found that the signals of both MCPP and P18 were mainly detected in the liver and tumors. However, the MCPP signal was stronger than the P18 signal in tumors (Figure 3B,C). In further experiments, we sliced the isolated tumor tissue and observed through confocal imaging that the MCPP signal was stronger than the P18 signal (Figure 3D,E), indicating the strong tumor‐selection ability of MCPP.

The tumor penetration of MCPP was measured using an in vitro CT26 MCS model. CT26 MCSs were incubated with MCPP, and the MCPP signals were detected by CLSM at different *Z*‐axis distances. After incubation for 2 h, CLSM showed a weak signal of MCPP in the peripheral area of the MCSs at *Z*‐axis distances of 60, 80, and 100 µm. After incubation for 6 h, CLSM showed an MCPP signal in the central area of the MCSs at a *Z*‐axis distance of 40 µm. After quantifying the intensity of the MCPP signal, we found that the intensity of MCPP significantly increased and reached its highest level at a *Z*‐axis distance of 40 µm after 12 h of incubation (Figure 3F,G). Therefore, these results show that MCPP exhibited high intracellular penetration.

### 2.5 Antitumor Effect of MCPP NPs via the Pyroptosis‐Evoked Immune Response

**Figure 4:**
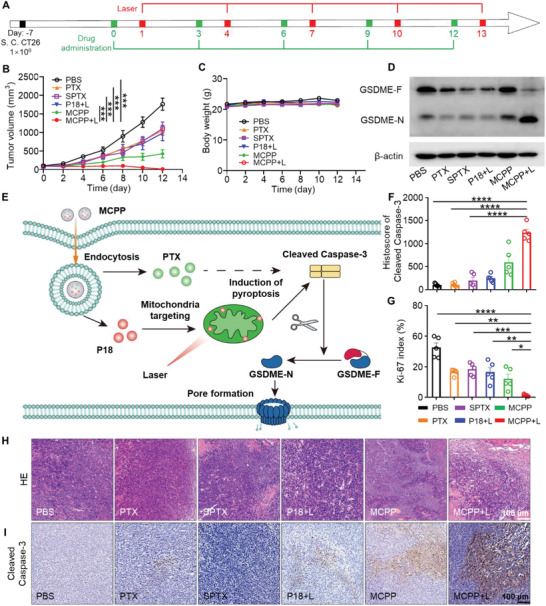
Antitumor effect and pyroptosis of MCPP. A) Treatment strategy for MCPP. B) The tumor growth curve of each group (*n* = 6). C) Body weight curve of each group (*n* = 6). D) Western blot detection of full‐length GSDME (GSDME‐F) and the GSDME‐N terminal domain (GSDME‐N). E) Schematic illustration of pyroptosis induced by MCPP+L. F) Quantitative analysis of the difference in cleaved Caspase‐3 expression on tumor slides (*n* = 5). G) Quantitative analysis of the difference in Ki‐67 expression on tumor slides (*n* = 5). H) Representative H&E stained tumor sections from each group. Scale bar = 100 µm. I) Representative image of cleaved Caspase‐3 immuno‐histochemistry staining of tumor sections from each group. Scale bar = 100 µm. (*p < 0.05, **p < 0.01, ***p < 0.001, and ****p < 0.0001).

Considering the favorable tumor‐targeting ability and antitumor ability of MCPP in vitro, we further evaluated the antitumor efficacy of MCPP in a CT26 tumor‐bearing BALB/c mouse model. The CT26 tumor model was established by subcutaneously injecting 1 × 10^6^ CT26 cells into the right flank of the mice. One week after CT26 tumor cell inoculation, the mice were randomly divided into six groups, and then, the mice were treated with PBS, free PTX, SPTX, free P18 with laser irradiation, MCPP and MCPP with laser irradiation (*n* = 6). All mice were administered an injection every 3 days via the tail vein, and laser irradiation was applied at 36 h after intravenous administration of free P18 and MCPP (**Figure** **4**A). The tumor volume and weight of mice were measured every 2 days. The results showed that the tumor volume of mice that received PBS rapidly increased after 6 days, whereas the tumor volumes of mice in the PTX, SPTX, and P18+L therapy groups were smaller than those of mice in the PBS group. MCPP exhibited a better ability to control tumors than PBS, PTX, and P18+L. MCPP+L showed the most effective tumor control capability. The tumors of mice that received MCPP+L therapy almost disappeared after 10 days (Figure 4B; Figures S24 and S25, Supporting Information). There were no significant changes in body weight in any of the groups (Figure 4C). We observed that tumor tissue was destroyed by MCPP+L therapy (Figure 4H), and that tumor cell proliferation was suppressed by MCPP+L therapy (Figure 4G; Figure S26, Supporting Information). These results indicated that MCPP+L treatment had the best ability to kill tumor cells.

**Figure 5:**
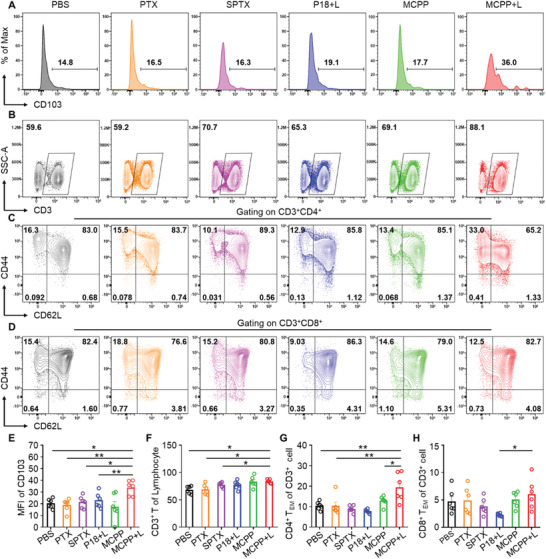
MCPP+L therapy initiates T‐cell clone expansion and differentiation. A) Flow cytometry revealed CD103 expression on lymph node DCs from each group, mean fluorscence intensity (MFI). B) Flow cytometry revealed the CD3^+^ T‐cell proportion in lymph nodes. C) Flow cytometry revealed CD44 and CD62L expressions on CD3^+^CD4^+^ T cells. D) Flow cytometry revealed CD44 and CD62L expressions on CD3^+^CD8^+^ T cells. E) Quantitative analysis of the difference in CD103 expression on lymph node DCs among each group (*n* = 6). F) CD3^+^ T‐cell proportion was increased in lymph nodes after MCPP+L therapy (*n* = 6). G) CD44^+^CD62L^−^CD4^+^ effector memory T cells (T_EM_) were increased after MCPP+L therapy (*n* = 6). H) CD44^+^CD62L^−^CD8^+^ effector memory T cells (T_EM_) were increased in the MCPP+L group compared with the P18+L group (*n* = 6). (*p < 0.05, **p < 0.01, ***p < 0.001, and ****p < 0.0001).

Pyroptosis induced by chemo‐based/photodynamic‐based therapy was executed by GSDME.^[^ ^26^ ^]^ GSDME is cleaved by Caspase‐3 and releases the GSDME‐N domain (GSDME‐N). GSDME‐N translocates into the cell membrane and forms membrane pores, which drive cell swelling, membrane rupture and DAMP release, resulting in a pyroptosis‐evoked immune response.^[^ ^26^, ^27^ ^]^ Therefore, we first detected GSDME in tumor tissue by western blot, and we found that the N‐terminal fragments of GSDME were increased after MCPP+L therapy. This result indicated that MCPP+L therapy could induce tumor cell pyroptosis (Figure 4D), and we also observed that cleaved Caspase‐3 expression was increased in the MCPP+L group (Figure 4F,I). Cleaved Caspase‐3 could activate GSDME, and the N‐terminal fragments of GSDME formed pores in the plasma membrane, leading to cell pyroptosis and the release of DAMPs (Figure 4E). To further confirm the pyroptotic effect, we conducted multiplex immuno‐histochemistry (mIHC) to detect CRT and HMGB1 in tumor tissues. We observed that MCPP+L increased CRT expression on the cell surface and released HMGB1 (Figure S27, Supporting Information). These results indicate that MCPP+L therapy can induce tumor cell pyroptosis. Tumor cell pyroptosis is favorable for inducing antitumor immune responses, and it has been reported that tumor cell pyroptosis could promote DC maturation and increase T‐cell clone expansion.^[^ ^8^, ^21^ ^]^ To investigate the immune effect in vivo, the lymph nodes were removed after the mice were euthanized, and we performed flow cytometry to detect the proportion of immune cells in the lymph nodes. We found that CD103 expression was upregulated on the DC surface after MCPP+L therapy (**Figure** **5**A,E). CD103 could serve as a bona fide DC marker and upregulation of CD103 indicates DC maturation.^[^ ^28^ ^]^ Therefore, MCPP+L therapy could induce DC maturation. After DC maturation, antigens were presented to T cells, initiating T‐cell clone expansion. We monitored the proportion of CD3^+^ T cells in the lymph nodes and found that the CD3^+^ T‐cell proportion was increased in the MCPP+L therapy group (Figure 5B,F), which indicated that MCPP+L therapy could promote T‐cell clone expansion. DC maturation and T‐cell clone expansion indicated that an adaptive immune response was initiated by the MCPP+L therapy. The generation of an immune memory effect is a consequence of an adaptive immune response. To explore whether MCPP+L therapy could generate memory T cells, CD44 and CD62L expressions on T cells were used to detect central memory T cells (T_CM_) and effector memory T cells (T_EM_). We found that CD4^+^ T_EM_ increased after MCPP+L therapy (Figure 5C,G). Moreover, CD8^+^ T_EM_ was also increased in the MCPP+L group compared with that in the P18+L group (Figure 5D,H). Based on these results, MCPP+L therapy could induce DC maturation, initiate T‐cell clone expansion, and trigger adaptive immunological responses.

### 2.6 MCPP+L Therapy Enhances the PD‐1 Blockade Efficiency and Triggers an Immunological Memory Response

**Figure 6:**
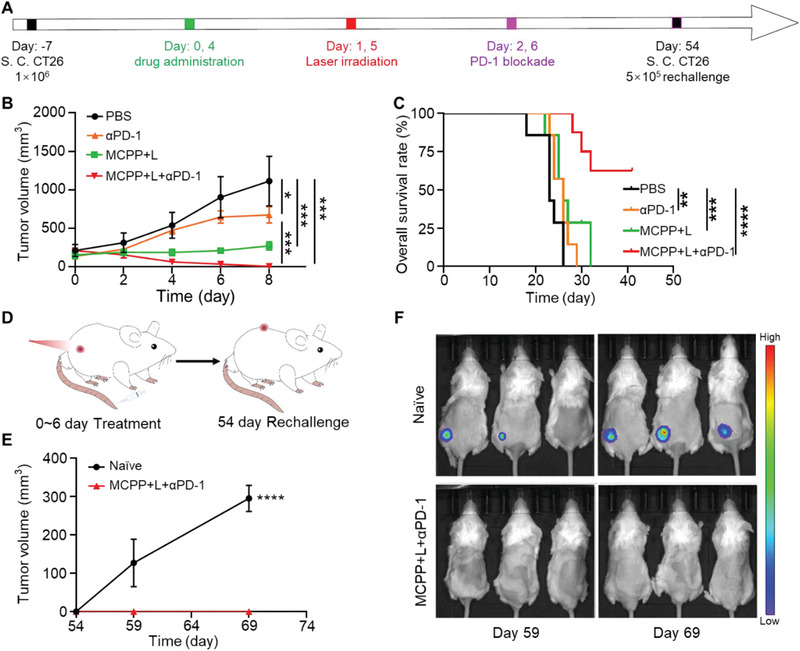
MCPP+L+anti‐PD‐1 therapy prevent tumor recurrence. A) Tumor inoculation and treatment strategy. B) Tumor growth curve of each group, anti‐PD‐1 (*α*PD‐1) (*n* = 7). C) Overall survival rate of mice in each group. D) Schematic illustration of the rechallenged experimental design (*n* = 7). E) Tumor growth curve of the naïve and rechallenged groups (*n* = 3). F) In vivo bioluminescence images monitoring tumor recurrence/growth in naïve and rechallenged groups. (*p < 0.05, **p < 0.01, ***p < 0.001, and ****p < 0.0001).

Tumor cell pyroptosis releases DAMPs to increase the efficiency of immune checkpoint blockade therapy.^[^ ^21^ ^]^ Therefore, we investigated whether MCPP could boost the efficacy of anti‐PD‐1 therapy. A CT26 tumor model was established by subcutaneously injecting 1 × 10^6^ CT26 cells expressing firefly luciferase (CT26‐Luc) into the right flank of mice. One week after CT26 tumor cell inoculation, the mice were randomly divided into four groups: PBS, anti‐PD‐1, MCPP+L, and MCPP+L+anti‐PD‐1 (*n* = 7, **Figure** **6**A). MCPP administration and laser irradiation were performed as described previously. PD‐1 antibody was administrated by intraperitoneal injection at 24 h after laser irradiation. The tumor size was monitored every day, and we observed that the tumor volume was increased in the PBS and anti‐PD‐1 groups. The tumor volume was stable in the MCPP+L group. MCPP+L combined with PD‐1 blockade therapy not only arrested tumor growth, but also induced massive tumor regression (Figure 6B). Moreover, in this group, three mice achieved tumor‐free survival, and all the mice survived for 30 days, thus showing a better outcome than with PBS, MCPP+L, and anti‐PD‐1 groups (Figure 6C), indicating that MCPP+L could act as an immune adjuvant to increases anti‐PD‐1 efficiency.

As a consequence of adaptive immunity, immunological memory responses are able to offer protection when the body re‐encounters pathogens. We also proved that MCPP+L therapy could generate memory T cells. We then assessed the immunological memory responses of the MCPP+L therapy. Three tumor‐free mice were selected from the MCPP+L+anti‐PD‐1 group, and 5 × 10^5^ CT26‐Luc cells were subcutaneously injected into the left flank of mice at 48 days after the primary tumor was removed by MCPP+L+anti‐PD‐1 therapy. Three same old naïve mice were also subcutaneously injected with 5 × 10^5^ CT26‐Luc cells in the left flank (Figure 6D). Fluorescence living imaging was used to monitor the rechallenged tumor. We observed that all naïve mice suffered from tumors, while all the mice that received MCPP+L+anti‐PD‐1 therapy were resistant to rechallenge after 15 days (Figure 6E,F), indicating that MCPP+L+anti‐PD‐1 therapy generated an immunological memory response. Therefore, MCPP could trigger an immunological memory response and prevent tumor recurrence.

### 2.7 Biosafety of MCPP

After the in vivo antitumor study, we performed in vivo safety experiments to verify the safety of the drug at the same dosage as that used in the treatment group. A total of 25 ICR mice were randomly divided into five groups and treated with PBS, free PTX, SPTX, free P18, and MCPP via the tail vein once every 3 days for a total of four times.Free PTX, SPTX, and MCPP were administered at a PTX dose of 10 mg kg^−1^. Free P18 was injected into tail vein at a dose of 0.8 mg kg^−1^. All mice were euthanized at 12 days, and peripheral blood was collected for routine blood and blood biochemical tests. The major organs were collected and sliced for pathological examination (Figures S28 and S29, Supporting Information). All tests and examinations suggested good biocompatibility of the MCPP.

## 3 Conclusions

In summary, we designed a novel tumor microenvironment ROS/GSH dual‐responsive nanoplatform to unleash antitumor immune response by inducing pyroptosis, boosting the efficiency of immune checkpoint blockade and achieving tumor growth control. We propose an innovative concept of the pyroptosis index to evaluate pyroptosis‐inducing ability. MCPP loaded with the cytotoxic agent PTX and phototoxicity agent P18 exhibited an excellent pyroptosis‐inducing capacity. Moreover, the ROS generated by P18 after laser irradiation achieved controlled drug release. The well‐designed structure endowed the MCPP system with high tumor retention and enhanced deep penetration into tumors, which were beneficial for inducing tumor‐specific pyroptosis. Furthermore, DAMPs released after tumor cell pyroptosis could promote DC maturation, initiate T‐cell clone expansion, prime adaptive antitumor efficiency, and boost anti‐PD‐1 efficiency, thus generating obvious immunological memory and prolonging survival time. Therefore, MCPP‐triggered chemo‐photodynamic therapy is a novel strategy for treating tumors and may be a promising immune adjuvant to boost anti‐PD‐1 efficiency via its robust pyroptosis‐inducing ability.

## 4 Experimental Section

### Materials

2‐(Methylthio)ethanol (99%), acryloyl chloride (96%,), 4‐dimethylaminopyridine (DMAP, 99%), triethylamine (TEA, 99.5%,), 2‐mercaptoethanol (99%), methacryloyl chloride (MA, 95%), 3‐(ethyliminomethyleneamino)‐*N*,*N*‐dimethylpropan‐1‐amine hydrochloride (EDC‐HCl, 99%), MTT, MPEG_5k_—NH_2_, CPPA—NHS (95%), 2,2″‐azobis(2‐methylpropionitrile) (AIBN, 98%), 3,3″‐dioctadecyloxacarbocyanine perchlorate (Dio, 98%), PTX (99%), and 3,3′‐dithiodipropionic acid (BCD, 99%) were all purchased from Sigma–Aldrich, China. Dulbecco's modified Eagle's medium (DMEM, 1×), RPMI 1640, FBS, penicillin streptomycin (PS), TrypLE Express (1×), 1× PBS (7.4), Alexa Fluor488 phalloidin (AF‐488), thiophene‐2‐thiol,4,6‐dia‐midino‐2‐10 phenylindole (DAPI), LIVE/DEAD viability kit, LysoTracker Green DND‐99, Mito Tracker Green FM, DPBF, reactive oxygen species assay kit (DCFH‐DA) were obtained from Life Technologies, China. The hematoxylin‐eosin (H&E) staining kit and the Ki‐67 staining kit were purchased from the Beyotime Biotechnology (Shanghai, China).The following immuno‐histochemistry, immunofluorescence, and immuno‐histochemical antibodies were used: Calreticulin (12238T, Cell Signaling Technology, CST), HMGB1 (6893S, CST), anti‐CD45‐PC5.5 (30‐F11, Invitrogen), cleaved Caspase‐3 (9961S, CST), anti‐CD3‐FITC (17A2, Biolegend), anti‐CD4‐ef450 (RM4‐5, Invitrogen), anti‐CD8‐PC5.5 (53‐6.7, Biolegend), anti‐CD44‐PE (IM7, Invitrogen), anti‐CD62L‐APC (MEL‐14, eBioscience), anti‐CD11b‐FITC (M1/70, Biolegend), anti‐Ly6C‐APC (HK1.4, Invitrogen), anti‐Ly6G‐PE (1A8, Biolegend), anti‐CD11C‐FITC (N418, Invitrogen), anti‐MHC II‐PC7 (M5/114.15.2, Biolegend), anti‐MHC‐II‐PE (M5/114.15.2, eBioscience) anti‐CD80‐PE (16‐10A1, Invitrogen), anti‐CD86‐APC (GL1, Invitrogen), and anti‐CD103‐PC7 (2E7, Biolegend).

### Synthesis of Monomer 2 (M2)

2‐(Methylthio) ethanol (10 mmol) was dissolved in dry 10 mL of dichloromethane (DCM) at ice bath; following that TEA (12 mmol) and DMAP (0.40 mmol) were added to the flask with stirring under argon atmosphere.Then acryloyl chloride (10 mmol) was added and the flask kept stirring for 12 h in dark. After the reaction was finished, the reaction solution was washed twice by saturated sodium chloride solution, and the organic layer was collected and dried by anhydrous Na_2_SO_4_, then filtrated and concentrated. Lastly, obtained colorless liquid was M2.

### Synthesis of Monomer 3 (M3)

Above obtained M2 and 2‐mercaptoethanol (10 mmol) were dissolved in 20 mL of anhydrous acetonitrile. Then TEA (10 mmol) was added dropwise, and the flask was placed in a 40 °C oil bath and continued reacting for 24 h. Next, the resulting mixture was concentrated by rotary evaporation, and the obtained light yellow oily liquid was M3.

### Synthesis of the Thioether Monomer 4 (M4)

First, the above‐obtained M3 (20 mmol) was dissolved in 30 mL of dry DCM; following, a few drops of TEA were added into the flask. Then, 10 mL of dry DCM contained MA (24 mmol) was added dropwise in an ice bath for 30 min. After that, the flask was placed at 25 °C for another 12 h. After removing the DCM, the residue was further purified by silica gel chromatography, which used hexane/ethyl acetate (v/v = 4:1) as eluent to obtain the final thioether M4.

### Synthesis of Polymer MPEG–CPPA

CPPA—NHS (0.13 mmol) and MPEG—NH_2_ (0.13 mmol) were dissolved in 3 mL of dry DCM, then a few drops of TEA were added into the flask and reacted for 48 h at 25 °C. After that, the product was purified by cold diethyl ether for three times and dried in a vacuum drying oven. Finally, the obtained pink solid powder was polymer MPEG–CPPA.

### Synthesis of Copolymer MPEG–CPPA‐*b*‐P(M4) (MPEG‐ROS)

MPEG–CPPA and AIBN were used as the RAFT agent and initiator, respectively, for the RAFT copolymerization. Specifically, MPEG–CPPA (0.01 mmol), M4 (1.00 mmol), and AIBN (0.005 mmol) were all dissolved in 3 mL of anhydrous Dio at a 25 mL Schlenk tube under argon atmosphere. Then, the mixture solution was subjected to three freeze–pump–thaw cycles in order to remove water and oxygen. Next, the tube was placed at 70 °C for 24 h. After the reaction was finished, the product was purified by cold diethyl ether for three times and dried in a vacuum drying oven. Finally, the obtained pink viscous solid was copolymer MPEG–CPPA‐*b*‐P(M4).

### Synthesis of SPTX

The GSH‐cleavable disulfate linker was synthesized as follows. Briefly, PTX (0.35 mmol) was dissolved in 5 mL of dry DCM; following, BCD (0.16 mmol), EDC‐HCl (0.77 mmol), and DMAP (0.05 mmol) were added in sequence and stirred for 1 h at 25 °C. Then above flask was added into another EDC‐HCl (0.37 mmol) and DMAP (0.05 mmol), and reacted for 24 h at 25 °C. Next, the obtained product was concentrated and further purified by silica gel chromatography, which used DCM/ethyl acetate (v/v = 2:1). Finally, the collected white solid power was SPTX.

### Preparation of MCPP NPs

Above obtained MPEG–CPPA‐*b*‐P (M4) was used as amphiphilic copolymer to together encapsulate hydrophobic SPTX and P18 through hydrophilic–hydrophobic interaction. Briefly, 2 mL dimethyl sulphoxide (DMSO) solution of SPTX (1 mg), P18 (1.0 mg), and MPEG‐CPPA‐*b*‐P (M4) (10 mg) was slowly added into 5 mL water under vigorous stirring at room temperature.After 0.5 h, the unloaded SPTX, P18, and DMSO were removed by dialysis method (MWCO = 3500 Da) against deionized (DI) water (1 L × 3). Finally, the MCPP NPs were prepared. The drug‐loading contents of SPTX and P18 in MCPP NPs were determined by a UV–vis spectrophotometer.

### H_2_O_2_/GSH‐Triggered Decomposition of MCPP

Copolymer carrier containing thioethers and disulfide‐linked SPTX endowed the MCPP NPs with H_2_O_2_/GSH dual‐responsive sustained drug release. The TEM images were used to observe the process of drug release. Briefly, the MCPP NPs were treated with 10 × 10^−3^ m GSH and 10 × 10^−3^ m H_2_O_2_ for 2 h, respectively. Then the morphology of MCPP NPs before and after treatment was observed by a transmission electron microscope (JEM‐1230EX).

The MCPP micelles were transferred into dialysis bags (MWCO = 3500 Da), which were immersed in PBS (pH 7.4) and hydrogen peroxide solution (10 × 10^−3^ m H_2_O_2_) in a shaking culture incubator at 37 °C. At different time points, 1.0 mL of sample solution was extracted from the dialysate and fresh corresponding buffer solution (1.0 mL) was added immediately. UV detection was performed in all samples to calculate the upload amount of the P18.

### Generation of ROS In Vitro

Singlet oxygen (^1^O_2_) production in vitro was determined using a DPBF probe. Briefly, MCPP micelles (P18: 5 µg mL^−1^) were mixed with DPBF (1 mg mL^−1^), and the mixture was treated with laser irradiation (1 W cm^−2^, 10 min) at 660 nm. Then, the mixture was collected for detection of the UV–vis spectrum every minute using a spectrophotometer (Shimadzu UV‐1800).

### Cell Culture and Animals

CT26 (mouse colon carcinoma) cancer cell line was purchased from the Shanghai Cell Bank of the Chinese Academy of Sciences and cultured in DMEM medium containing 10% FBS and 1% penicillin/streptomycin (P/S), with 5% CO_2_ at 37 °C. Additionally, all mice were obtained from Hubei Provincial Laboratory Animal Public Service Center and fed at specific pathogen‐free conditions at Wuhan University. All animal assays were approved by the Institutional Animal Care and Use Committee of Wuhan University. Animal experiments were carried out when the tumor volume reached ≈100–200 mm^3^.

### Cell Internalization and Localization Study

CT26 cells were cultivated in an 8‐well plate (3 × 10^4^ cells per well) and incubated with MCPP NPs (P18: 5 µg mL^−1^) for 2 and 6 h. The cells were then fixed with 4% paraformaldehyde, bovine albumin (BSA), and X‐100 in sequence.The cell cytoskeleton and nuclei were stained with AF‐488 and DAPI, followed by the observation using CLSM (Zeiss 800).

To investigate the colocalization effect of the MCPP NPs, CT26 cells were first incubated with MCPP NPs (P18: 5 µg mL^−1^) for 2 and 6 h, followed by staining of lysosomes and mitochondria with LysoTracker Green DND‐99 and MitoTracker Green FM, respectively. The colocalization of MCPP NPs was determined using CLSM. To investigate the lysosomal escape of the MCPP, CT26 cells were stained with LysoTracker Green DND‐99. The colocalization of P18 signal and lysosomes was detected by CLSM (Zeiss 800) at the indicated time points. Furthermore, the cellular uptake behavior of MCPP NPs was determined by flow cytometry (NovoCyte 2060R, USA). In a typical experiment, MCPP NPs (P18: 5 µg mL^−1^) were incubated with CT26 cells (1 × 10^5^ cells per well) for 10 min, 30 min, and 1 h. At different time points, CT26 cells were washed with PBS three times. Subsequently, cells were treated with trypsin, suspended in DMEM, and centrifuged at 3000 rpm for 5 min. The cell pellets obtained were re‐suspended in PBS (0.4 mL). The percentage of cells associated with MCPP NPs was assessed using flow cytometry.

### Intracellular Generation of ROS

Intracellular ROS generation of MCPP NPs was determined by using ROS detection kit (DCFH‐DA). CT26 cells were cultivated at 12‐well plate (5 × 10^4^ cells per well) and incubated for 6 h with PBS, free P18, and MCPP NPs (P18: 5 µg mL^−1^). The cells were then exposed to 660 nm laser irradiation (1 W cm^−2^, 3 min). After irradiation, the cells were incubated with DCFH‐DA probe (10 × 10^−6^ m L^−1^) for another 20 min. The cells without irradiation treatment were used as control group. The fluorescence images of cells were examined by CLSM (Zeiss 800). ROS generation of MCPP NPs was also tested in CT26 MCSs, which experimental procedure was same as above.

### In Vitro Cytotoxicity Studies

For the cytotoxicity assay, 200 µL of fresh DMEM containing CT26 cells was seeded into 96‐well plates (1 × 10^4^ cells per well) and cultivated overnight. Then, the used DMEM was replaced with fresh DMEM containing different concentrations of P18 or PTX (P18: 0, 0.008, 0.04, 0.08, 0.4, and 0.8 µg mL^−1^; PTX: 0, 0.1, 0.5, 1, 5, and 10 µg mL^−1^), and the cells were treated for 24 h. The cells were then treated with a 660 nm laser (1.0 W cm^−2^) irradiation for 3 min and cultivation was continued for another 24 h. The medium was then removed, and MTT was added to each well. After 4 h of incubation, MTT was removed, and 200 µL of DMSO was added to each well to dissolve the violet formazan crystals produced. The fluorescence of cells at 570 nm was measured using a SPARK‐10 M microplate reader (Tecan).

### Live/Dead Cell Staining Study

For the live/dead staining assay, CT26 cells and CT26 MCSs (3D) were used to further evaluate the chemotherapy and photodynamic efficacy of MCPP NPs in vitro. Briefly, CT26 cells were seeded into 12‐well plates (1.5 × 10^5^ cells per well) and incubated overnight. Different formulations were added to each well (P18: 2.4 µg mL^−1^; PTX: 30 µg mL^−1^) for 24 h. Moreover, P18 and MCPP groups were treated with or without 660 nm laser irradiation (1.0 W cm^−2^) for 3 min. The group with no treatment was used as the control. Next, the cells were stained with fluorescein diacetate (FDA)/propidium Iodide (PI) for 20 min in the dark, washed gently with PBS, and subsequently imaged using a fluorescence microscope (Olympus, IX 73).In addition, CT26 MCSs were cultivated with the above different formulations for 6 h with or without 660 nm laser irradiation (1.0 W cm^−2^) for 3 min. The subsequent steps were similar to those described above.

### In Vitro Pyroptosis Index Evaluation

CT26 cells were seeded into 6‐well plates (5 × 10^4^ cells per well) and incubated for 36 h. Then a different formulation was added in each well (P18: 2.4 µg mL^−1^; PTX: 30 µg mL^−1^) for 48 h. Moreover, P18+L and MCPP+L groups were treated with a 660 nm laser irradiation (1.0 W cm^−2^) for 3 min after P18 and MCPP were added in each well for 1 h. Cell morphology was observed using a microscope (Leica) at 12, 24, 36, and 48 h after treatment, and the balloon‐like cells were considered pyroptotic cells. The ratio of the pyroptotic cell number to the total cell number in a snapshot multiplied by 100 was applied as the pyroptosis index of each treatment at the indicated time point.

### In Vitro LDH Release Detection

CT26 cells were seeded into 96‐well plates (5 × 10^4^ cells per well) and incubated for 36 h. Different formulations were added to each well (P18: 2.4 µg mL^−1^; PTX: 30 µg mL^−1^) for 12 h. Moreover, P18+L and MCPP+L groups were treated with a 660 nm laser irradiation (1.0 W cm^−2^) for 3 min after P18 and MCPP were added to each well for 1 h. Cell culture supernatant from each treatment was collected and added into a new 96‐well plate for LDH release detection. LDH levels in the cell culture supernatant were detected using the LDH release assay kit (Beyotime Biotechnology, C0016). The fluorescence of the cell culture supernatant at 490 or 600 nm was measured using PowerWave XS2.

### In Vitro CRT and HMGB1 Detection

CT26 cells were seeded into confocal dishes (5 × 10^4^ cells per well) and incubated for 36 h. Different formulations were added to each well (P18: 2.4 µg mL^−1^; PTX: 30 µg mL^−1^) for 12 h. P18+L and MCPP+L groups were treated with a 660 nm laser irradiation (1.0 W cm^−2^) for 3 min after P18 and MCPP were added to each well for 1 h. CT26 cells were fixed with 4% paraformaldehyde and permeabilized with 0.1% Triton X‐100. The cells were then incubated with anti‐CRT or anti‐HMGB1 antibodies and fluorescein isothiocyanate (FITC)‐conjugated secondary antibodies.The cells were then counterstained with DAPI. CRT and HMGB1 fluorescence were detected using CLSM.

### Deep Penetration Study

CT26 multicellular spheroids (MCSs) were prepared using a previously reported method.Briefly, CT26 cells were seeded in 96‐well plates (1 × 10^3^ cells per well) containing agarose and then cultured for 3–5 days to obtain the MCSs. The MCPP NPs (P18: 10 µg mL^−1^) were incubated with MCSs for 2, 6, or 12 h. The MCSs were then washed twice with PBS and stained with DAPI. Finally, the fluorescence signals of the MCSs were observed using a confocal microscope.

### Body Distribution Evaluation

Female BALB/c mice were injected subcutaneously with 1 × 10^6^ CT26 cells per mouse. When tumors reached ≈200 mm^3^, all mice were randomly divided into two groups and intravenously injected with MCPP and P18 at a P18 dose of 0.8 mg kg^−1^. The signals of MCPP and P18 were detected by in vivo imaging at 12, 24, 36, 48, 72, and 96 h. At 96 h post injection, the mice were euthanized, and the major organs (heart, liver, spleen, lung, kidney, and tumor) were excised for ex vivo fluorescence imaging.

### Antitumor Effect Evaluation

Female BALB/c mice were injected subcutaneously with 1 × 10^6^ CT26 cells per mouse. 7 days later, when tumors reached 100 mm^3^, the mice were randomly divided into six groups (*n* = 6; PBS, PTX, SPTX, P18+L, MCPP, and MCPP+L), and were injected with PBS, free PTX, SPTX, free P18, and MCPP via tail vein once every 3 days for five times at a PTX dose of 10 mg kg^−1^ or at a P18 dose of 0.8 mg kg^−1^. Mice treated with free P18+L and MCPP+L received laser irradiation 36 h post injection. Tumor size and body weight were measured every 2 days. The tumor volume was calculated according to the following formula: width^2^ × length × 0.5. All groups of mice were euthanized 14 days after the start of treatment, and the tumors, inguinal lymph nodes, and spleen were excised. The tumors were weighed and photographed. They were then sliced for H&E and Ki‐67 staining.

### Multiplexed Immuno‐Histochemistry Staining and Scanning

Opal 7‐Color Manual IHC Kit (NEL811001KT; Waltham) was used in this step. Briefly, tumor tissues were excised and sliced as above. Tumor slides were antigen‐retrieved by AR6 buffer with microwave treatment, followed by incubation with antibodies (Calreticulin, 12238T, CST. HMGB1, 6893S CST) and tyramide signal amplification (TSA; PerkinElmer Opal kit). The microwave treatment step, antibody incubation, and TSA were repeated until the samples were incubated with the last antibody. Finally, DAPI was used for nuclear counterstaining. Slides were scanned using a PerkinElmer Vectra scanner (Scanned by Vectra 2.0.8.; PerkinElmer), and all scanned images were prepared and analyzed using the Inform 2.0.

### Immuno‐Histochemistry Staining

Tumor tissues were excised and sliced as described above, and all slides were antigen‐retrieved by citrate (pH 6.0) and then incubated with cleaved Caspase‐3 as primary antibody at 4 °C overnight. After incubation with the corresponding secondary biotinylated immunoglobulin G antibody solution and an avidin‐biotinperoxidase reagent, sections were stained with DAB kit and lightly counterstained with Mayer's hematoxylin. All slides were scanned using a Pannoramic MIDI Slide scanner (3D HISTECH).

### Western Blot

Western blot analysis was performed as previously reported.^[^ ^29^ ^]^ After each treatment, mouse tumors were lysed using radio immunoprecipitation assay (RIPA) reagent (Pierce, Rockford, IL, USA) containing a complete miniprotease inhibitor cocktail and phosphate inhibitors (Roche, Branchburg, NJ, USA).Proteins were separated using 10% sodium dodecyl sulfate (SDS)–polyacrylamide gel electrophoresis and transferred onto polyvinylidene fluoride membranes (Millipore).Then, the protein was incubated with anti‐GSDME (Abcam, ab215191) and anti‐*β*‐actin antibody (Abcam, ab8226).

### MCPP+L Therapy Combined with PD‐1 Blockade Therapy and Tumor Recurrence Prevention

Female BALB/c mice were injected subcutaneously with 1 × 10^6^ CT26 cells expressing firefly luciferase (CT26‐Luc) on the right flank of each mouse. When tumors reached 100 mm^3^, the mice were randomly divided into four groups (*n* = 7; PBS, anti‐PD‐1, MCPP+L, and MCPP+L+anti‐PD‐1) as described above, and anti‐PD‐1 (10 mg kg^−1^) was administered by intraperitoneal injection at 24 h after MCPP+L therapy. Anti‐PD‐1, MCPP+L, and MCPP+L+anti‐PD‐1 therapies were administered twice every 4 days. Tumor volume was monitored using a caliper every 2 days. When the tumor volume reached 2500 mm^3^, the mice were euthanized and documented as dead. Forty‐eight days after the last therapy, tumor‐free mice in the MCPP+L+anti‐PD‐1 group and naïve mice were injected with 5 × 10^5^ CT26‐Luc cells at the left flank. Tumor growth was monitored using fluorescence imaging.

### Flow Cytometry

The tumor, inguinal lymph nodes, and spleen were excised as described above. Tumor tissue was dissociated into homogenates using a GentleMACS Dissociator (130‐093‐235) and then enzymatically digested in a humidified incubator at 37 °C with 5% CO_2_ for 1 h. Single‐cell suspensions of tumor infiltrating lymphocytes (TILs) were collected using the density gradient centrifugation method.Inguinal lymph nodes and spleens were also processed into single‐cell suspension. The single‐cell suspension was then stained with anti‐CD45‐PC5.5 (30‐F11, Invitrogen), anti‐CD3‐FITC (17A2, Biolegend), anti‐CD4‐ef450 (RM4‐5, Invitrogen), anti‐CD8‐PC5.5 (53‐6.7, Biolegend), anti‐CD44‐PE (IM7, Invitrogen), anti‐CD62L‐APC (MEL‐14, eBioscience), anti‐CD11b‐FITC (M1/70, Biolegend), anti‐Ly6C‐APC (HK1.4, Invitrogen), anti‐Ly6G‐PE (1A8, Biolegend), anti‐CD11C‐FITC (N418, Invitrogen), anti‐MHC II‐PC7 (M5/114.15.2, Biolegend), anti‐MHC‐II‐PE (M5/114.15.2, eBioscience), anti‐CD80‐PE (16‐10A1, REF: 12‐0801‐81, Lot: 2186515, Invitrogen), anti‐CD86‐APC (GL1, Invitrogen), and anti‐CD103‐PC7 (2E7, Biolegend) according to the manufacturer's protocols. Flow cytometry was performed as previously described with an FACS Caliber flow cytometer equipped with CellQuest software (Beckman), and dead cells were excluded based on Fixable Viability Dye‐eFluor 506 (eBioscience). The samples were analyzed using FlowJo (Tree Star). The gating strategy of different immune cells is shown in Figures S30–S32 (Supporting Information).

### Biosafety Evaluation

A total of 25 ICR mice were randomly divided into five groups and treated with PBS, free PTX, SPTX, free P18, and MCPP via the tail vein once every 3 days for four times. Free PTX, SPTX, and MCPP were administered at a PTX dose of 10 mg kg^−1^ or a P18 dose of 0.8 mg kg^−1^, respectively. All mice were euthanized after 12 days, and peripheral blood was collected for routine blood tests and blood biochemical tests. Blood biochemical tests included alanine aminotransferase (ALT), aspartate aminotransferase (AST), creatinine (CERA), and urea (UREA), and routine blood tests included white blood cell count (WBC), lymphocyte ratio (LYM), red blood cell count (RBC), hemoglobin concentration (HGB), red‐blood‐cell‐specific volume (HCT), mean corpuscular hemoglobin content (MCH), red cell volume distribution width (RDW), and blood platelet counts (PTL). The major organs were collected and sectioned for pathological examination.

### Statistical Analysis

The experimental data were presented as mean ± standard error of the mean (SEM) using GraphPad Prism 8.0. The sample size (*n*) for each statistical analysis is provided in the figure legends. GraphPad Prism software was used to calculate the *p*‐value using the unpaired two‐sided Student's *t*‐test for comparison of difference between two groups or one‐way analysis of variance (ANOVA) followed by Turkey's multiple comparisons and two‐way ANOVA followed by Turkey's multiple comparisons for comparison of differences between more than two groups. The log‐rank (Mantel–Cox) test was used to compare the survival curves. Differences were considered statistically significant at *p*‐values less than 0.05. In all cases, statistical differences were considered at *p < 0.05, **p < 0.01, ***p < 0.001, ****p < 0.0001, not significant (ns), and *p* > 0.05.

### Ethics Statement

The animal experimental protocol was approved by the Experimental Animal Ethics Committee of the School and Hospital of Stomatology at Wuhan University (S07920070B).

## Conflict of Interest

The authors declare no conflict of interest.

## Supporting information

Supporting Information

## Data Availability

Research data are not shared.
