# Supplementary material for: Microenvironment‐Responsive Prodrug‐Induced Pyroptosis Boosts Cancer Immunotherapy
Source: Adv Sci (Weinh). 2021 Oct 27;8(24):2101840. doi: 10.1002/advs.202101840 (PMC8693073; doi:10.1002/advs.202101840)
Supplement: Supplementary file 1 — Supporting Information [file ADVS-8-2101840-s001.pdf]

© 2021 Wiley-VCH GmbH

ADVANCED SCIENCE Open Access

Supporting Information

for *Adv. Sci.*, DOI: 10.1002/advs.202101840

Microenvironment responsive prodrug induced pyroptosis boost cancer immunotherapy

Yao Xiao, Tian Zhang, Xianbin Ma, Qi-Chao Yang, Lei-Lei Yang, Shao-Chen Yang, Mengyun Liang, Zhigang Xu and Zhi-Jun Sun**

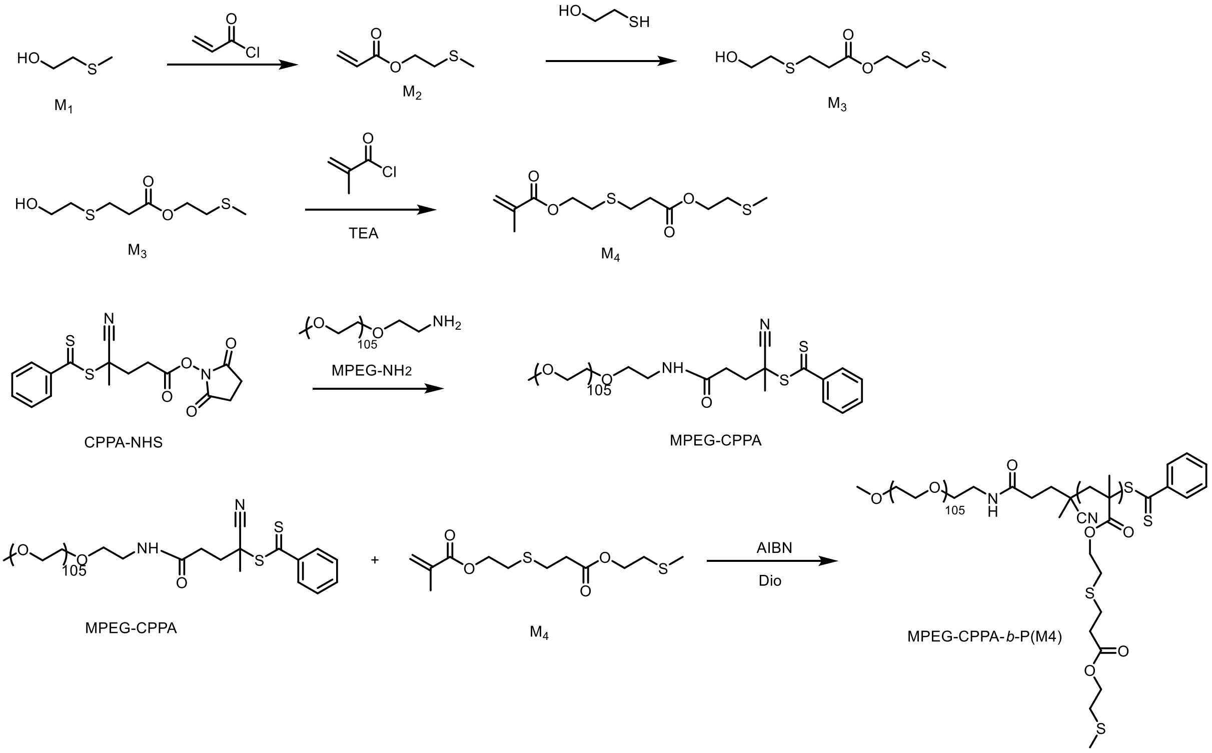

Figure S1. The synthesis routes of the thioether functional M4 and MPEG-CPPA-*b*-P (M4) copolymer.

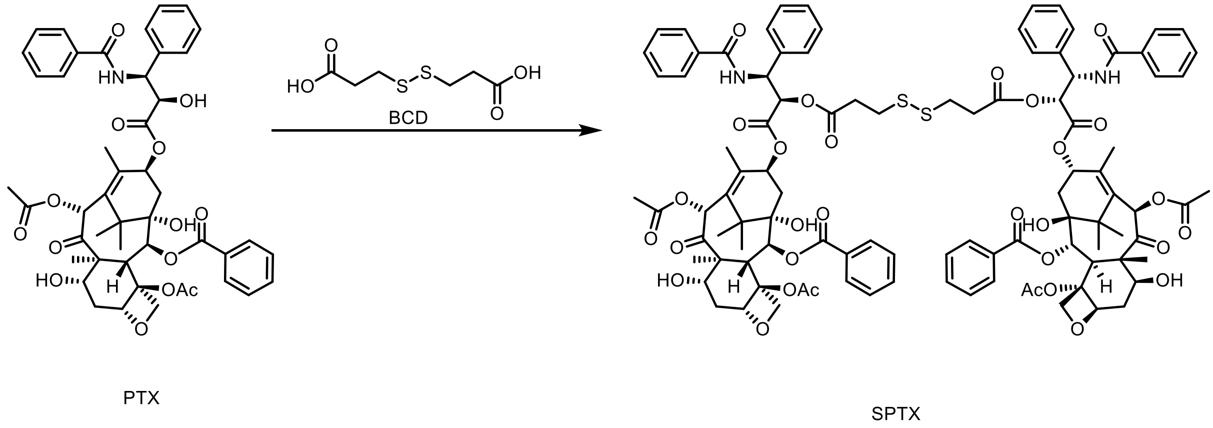

Figure S2. The synthesis route of SPTX.

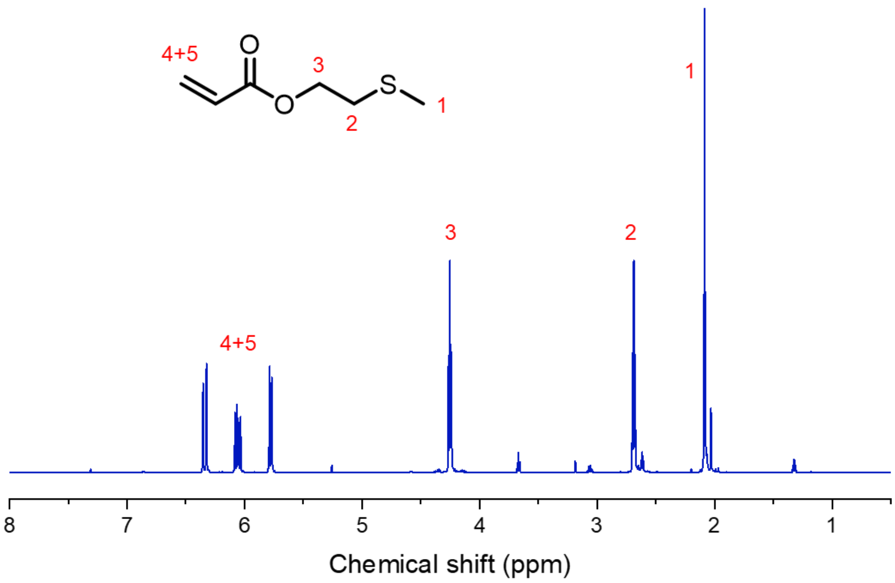

Figure S3. ^{1}H NMR spectrum of M2.

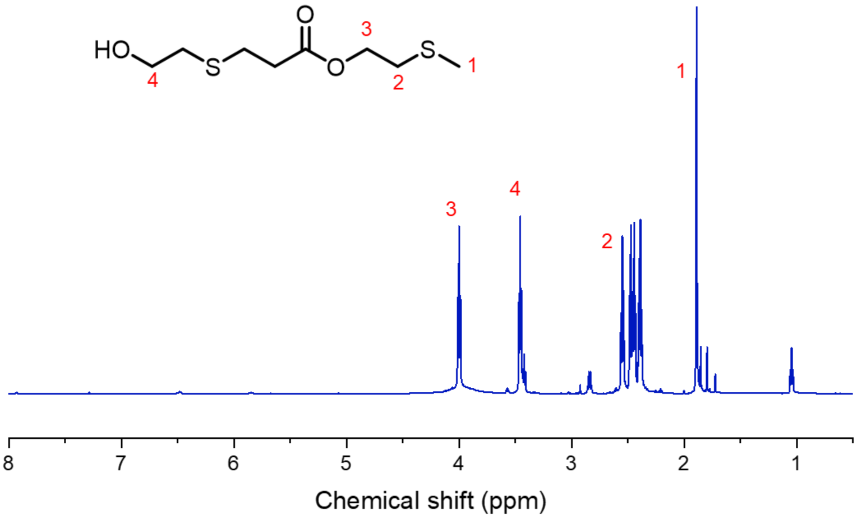

Figure S4. ^{1}H NMR spectrum of M3.

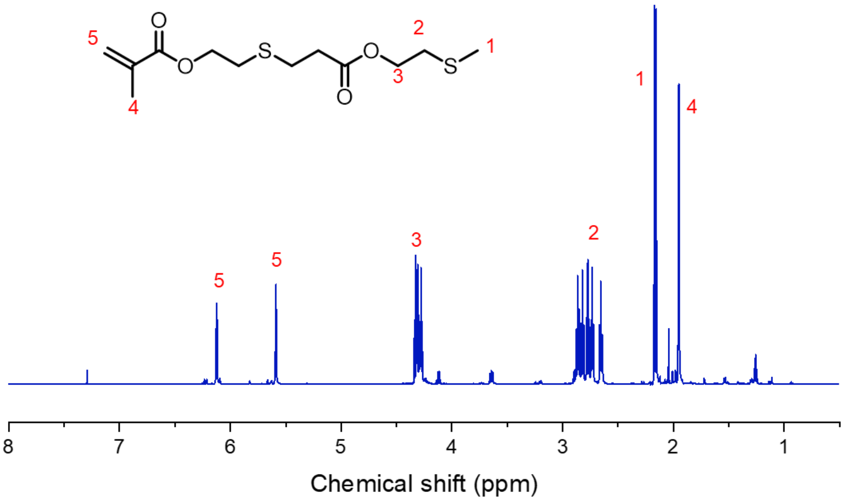

Figure S5. ^{1}H NMR spectrum of M4.

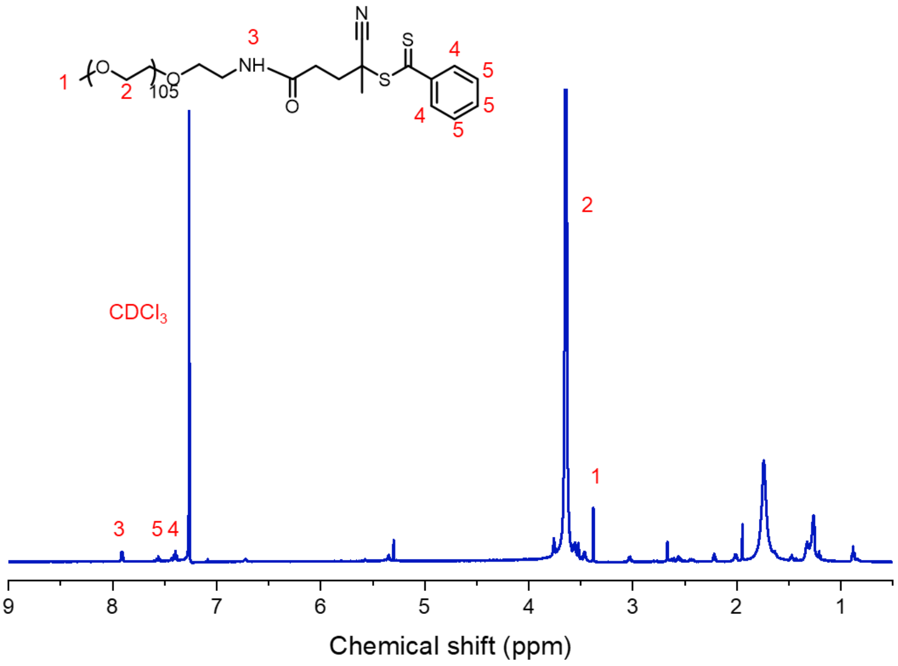

Figure S6. ^{1}H NMR spectrum of MPEG-CPPA.

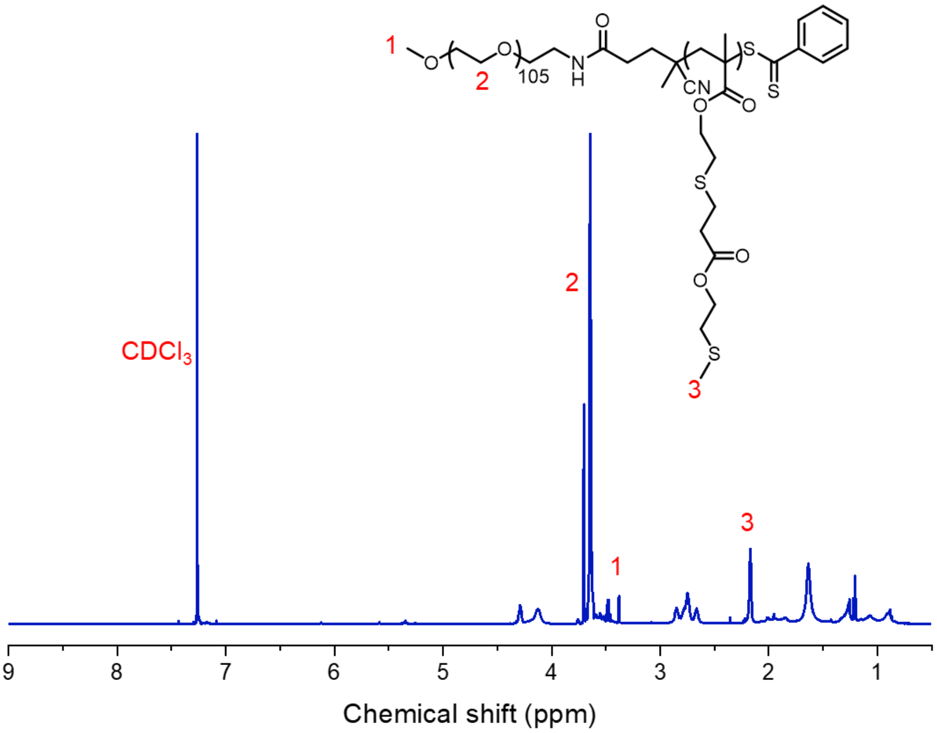

Figure S7. ^{1}H NMR spectrum of MPEG-CPPA-*b*-P (M4).

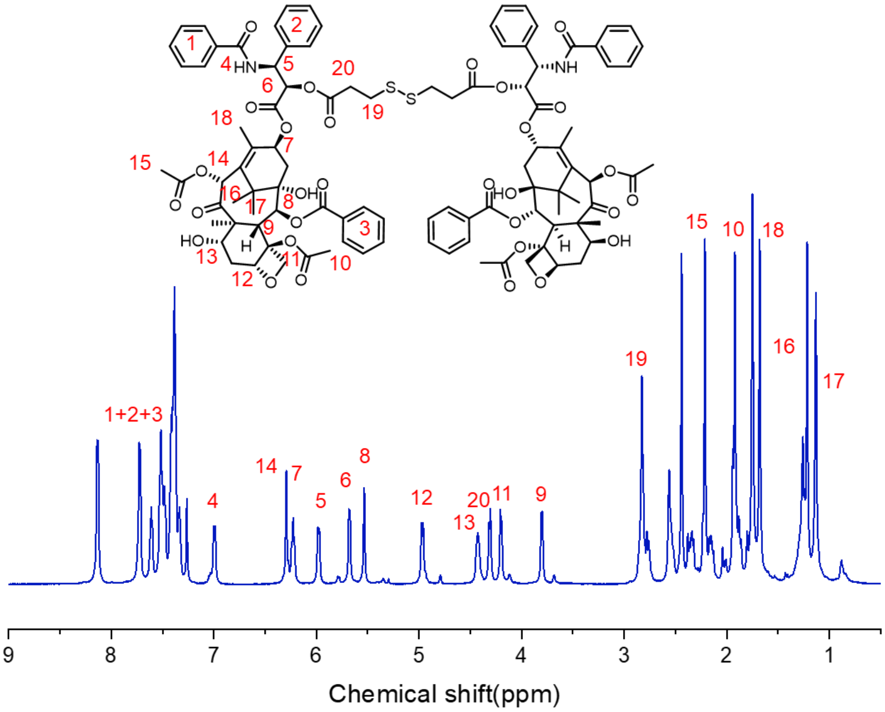

Figure S8. ^{1}H NMR spectrum of SPTX.

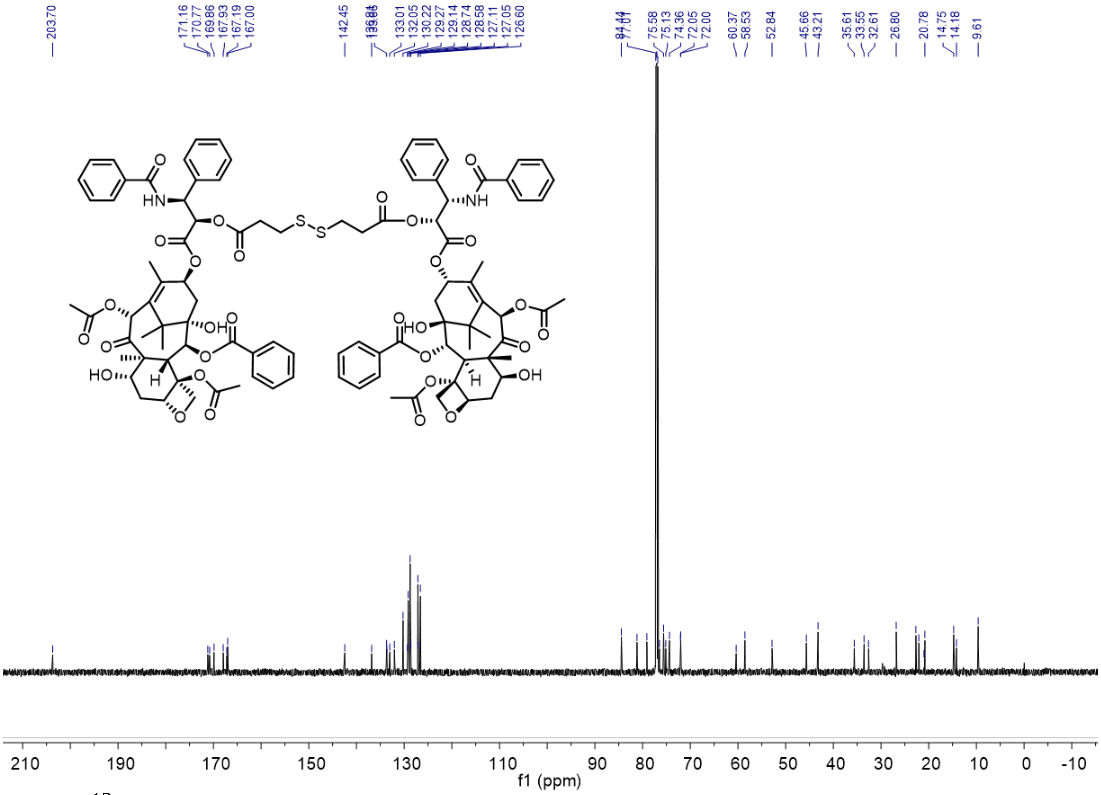

Figure S9. ^{13}C NMR spectrum (150 MHz) of SPTX in $CDCl_3$.

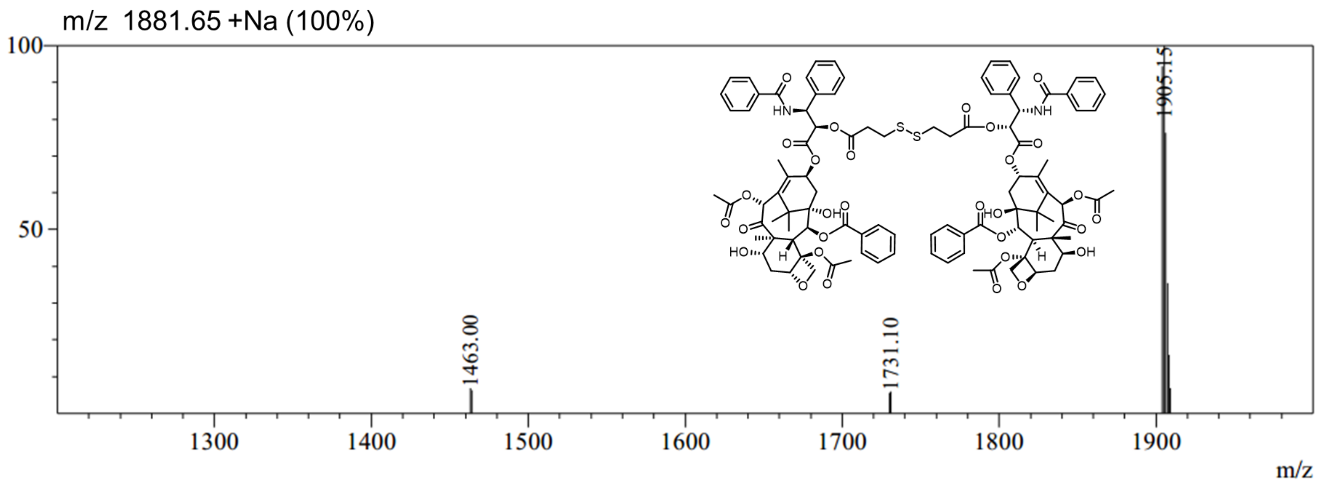

Figure S10. Mass spectrum of SPTX.

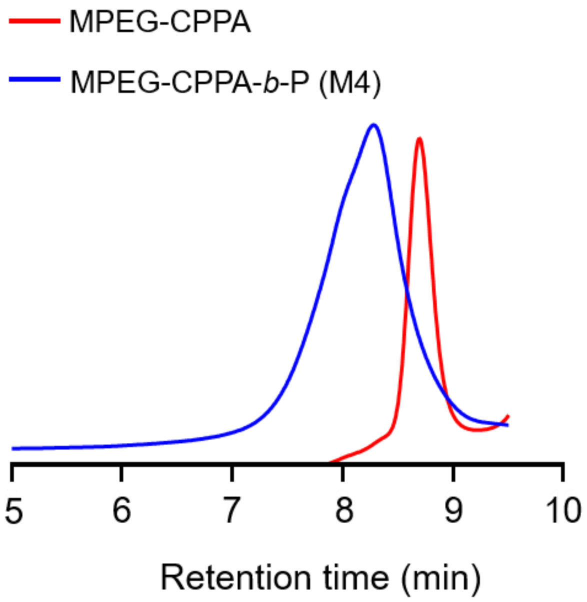

Figure S11. GPC traces of MPEG-CPPA and MPEG-CPPA-*b*-P (M4).

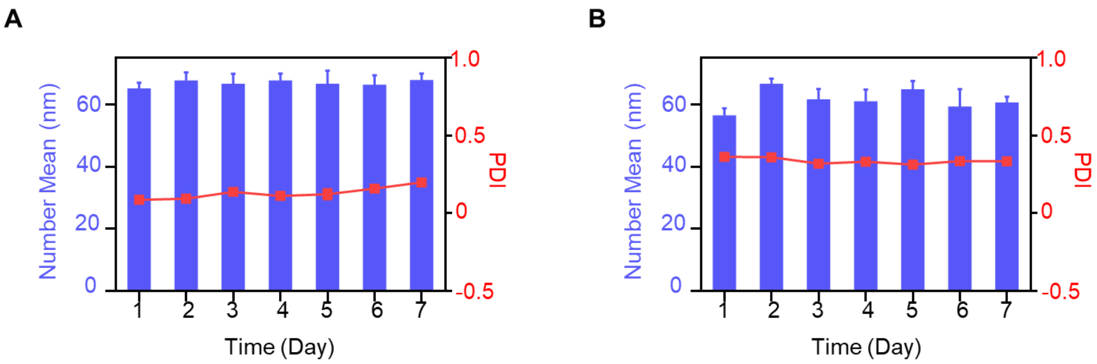

Figure S12. The stability of MCPP in RPMI (A) and 10 % FBS (B) (n = 3).

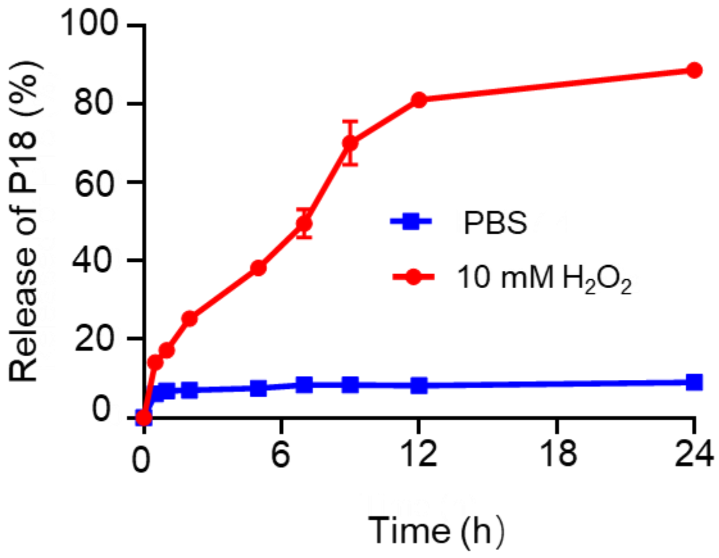

Figure S13. Cumulative release of P18 from PBS and 10 mM H_2O_2 solution.

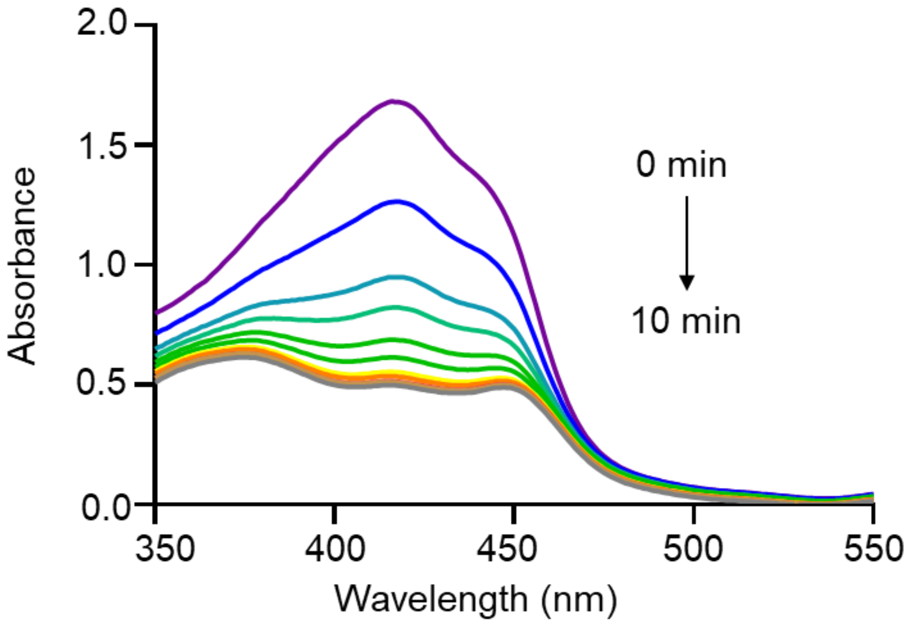

Figure S14. The UV absorbance spectra of MCPP micelles with DPBF probe (660 nm laser, 1.0 W/cm^2) for 10 min.

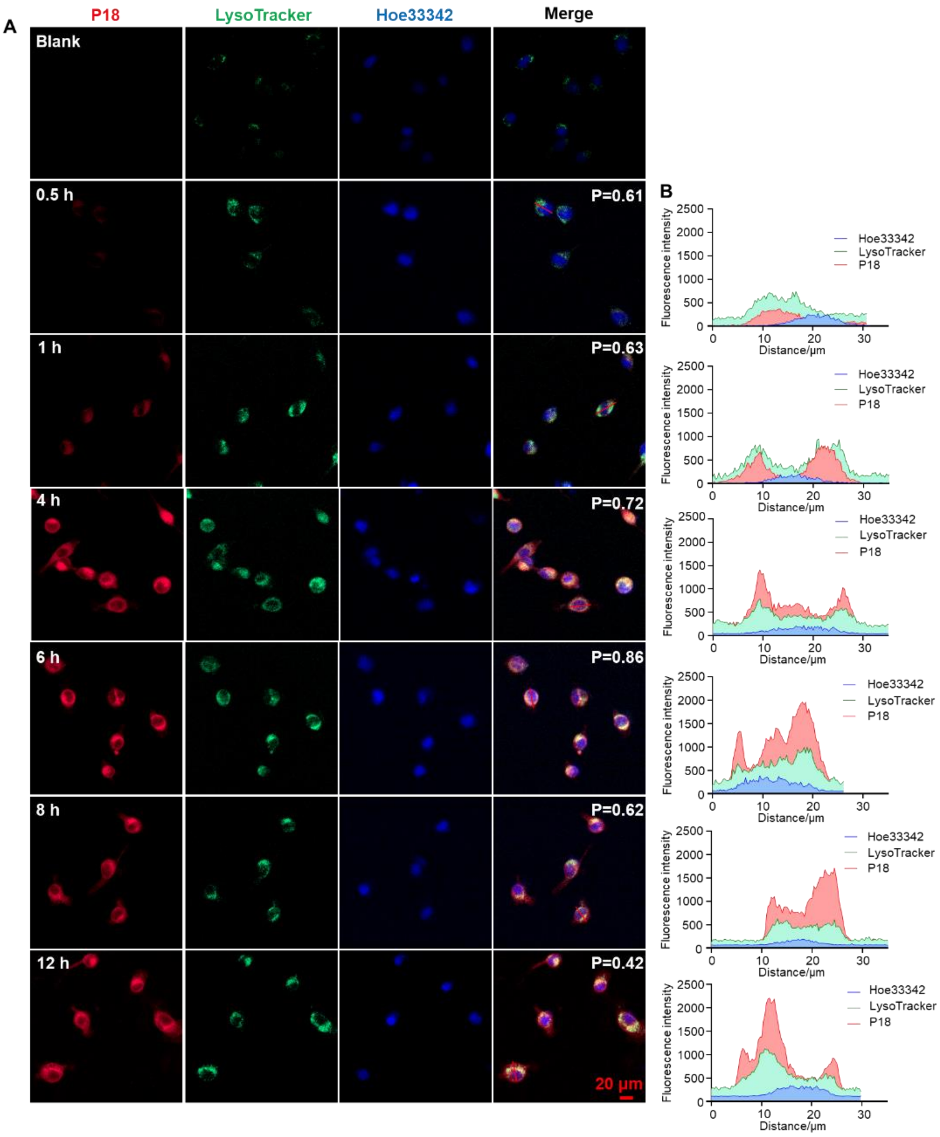

Figure S15. The lysosomal escape of the nano-prodrugs. Representative CLSM image (A) and quantitative analysis (B) of co-localization of MCPP with lysosome in CT26 cells. The lysosme (green) was marked by LysoTracker and nuclear (blue) stained bu Hoest33342. Scale bar = 20 μm.

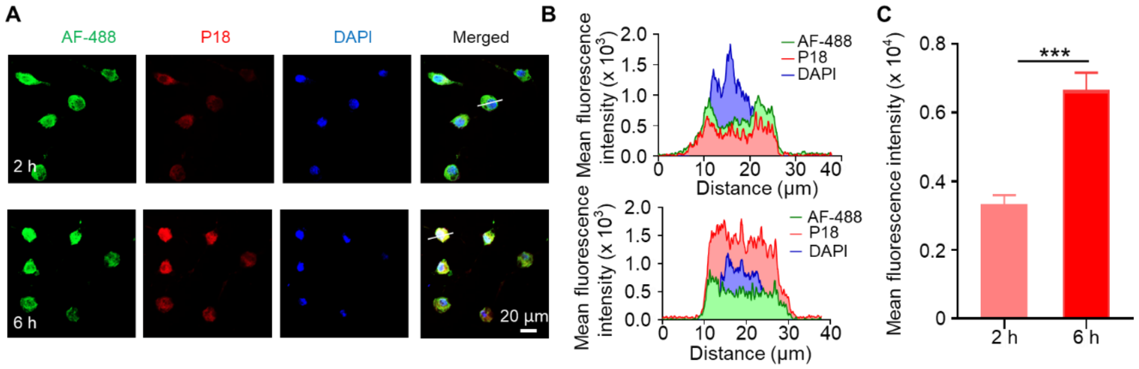

Figure S16. CLSM image (A) and quantitative analysis (B, C) of P18 signal in CT26 cells treated with MCPP for 2 h and 6 h (n = 3). The cytoskeleton (green) was marked by AF-488, and the nuclear (blue) was stained with DAPI. Scale bar = 20 μm. (*p < 0.05; **p < 0.01; ***p < 0.001; ****p < 0.0001)

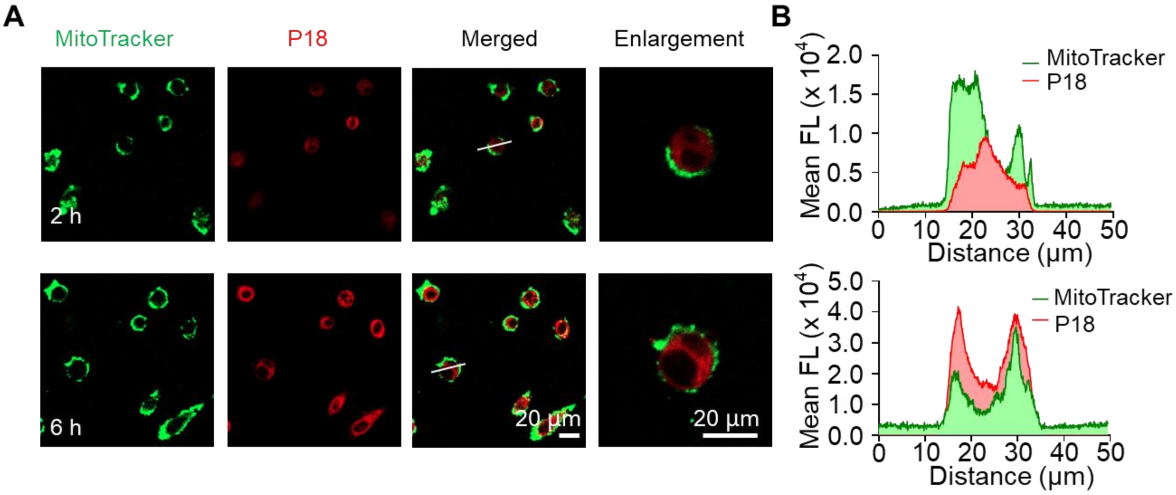

Figure S17. Intracellular trafficking and co-localization of P18 in CT26 cells. Representative CLSM image (A) and quantitative analysis (B) of co-localization of MCPP with mitochondrial in CT26 cells, CT26 cells were incubated with MCPP for 2 h and 6 h, then treated with MitoTracker (green fluorescence) for 15 min and observed by CLSM. Scale bar = 20 μm.

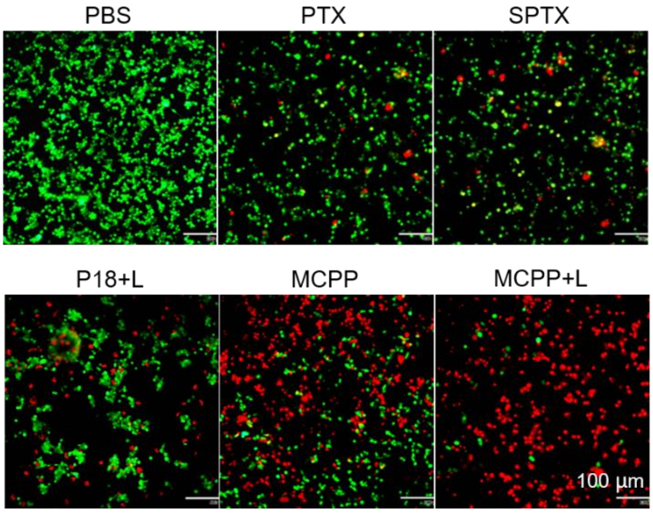

Figure S18. Fluorescence images of live (FDA, green signal) / dead (PI, red signal) CT26 cells stained by FDA (green) or PI (red). Scale bar = 100 μm.

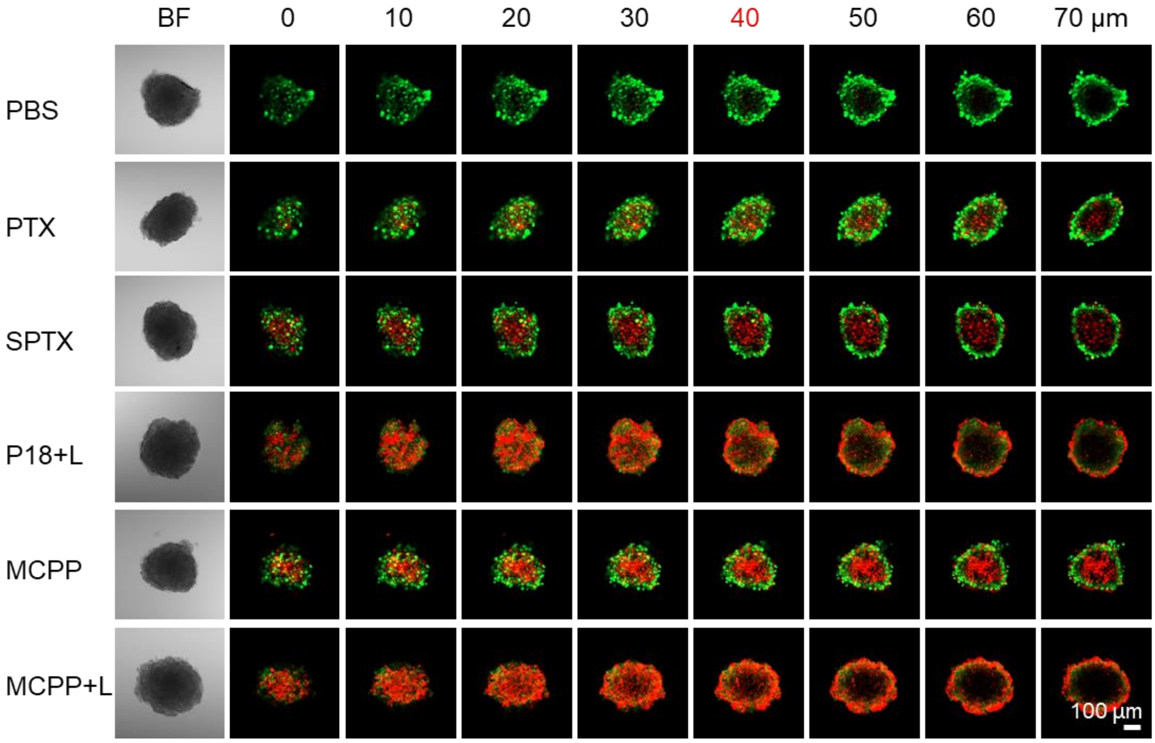

Figure S19. CLSM images of live (FDA, green signal) / dead (PI, red signal) assay by CT26 MCSs at different penetration depth to evaluation cytotoxicity among PBS, PTX, SPTX, P18+L, MCPP, and MCPP+L group. Bright field (BF), scale bar = 100 μm.

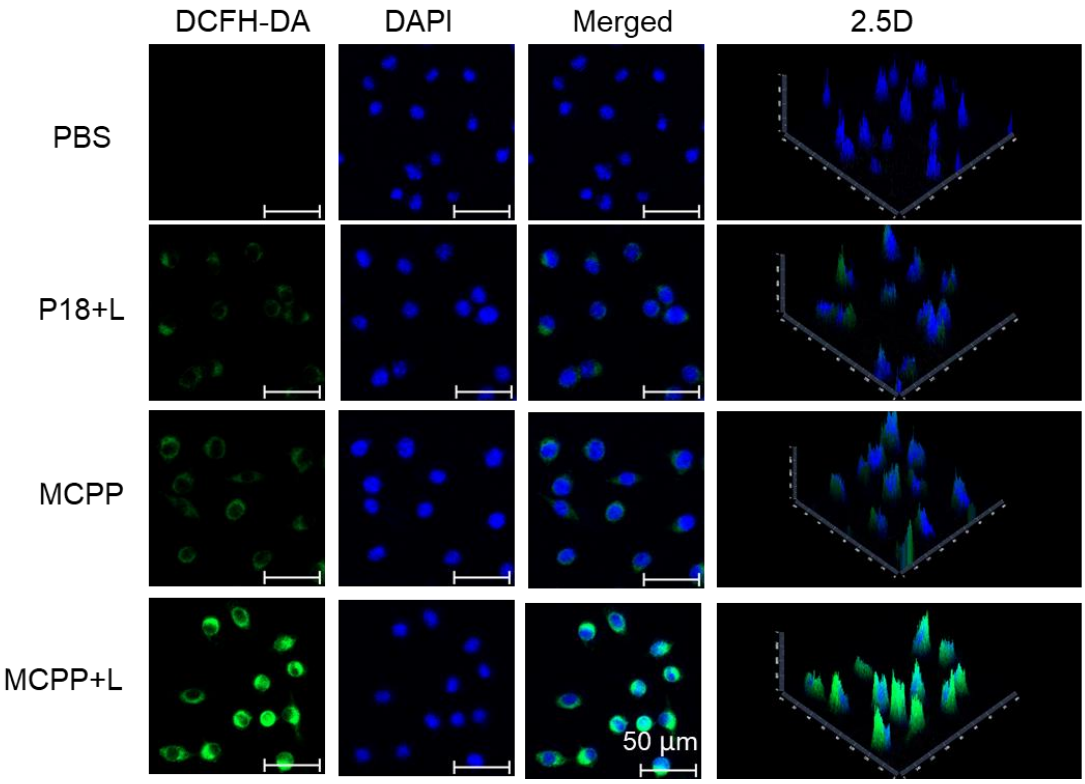

Figure S20. ROS detection in CT26 cells after treated with PBS, P18+L, MCPP, MCPP+L at a P18 dose of 5 μg/mL for 6 h, ROS was detected by DCFH-DA (green signal). Scale bar = 50 μm.

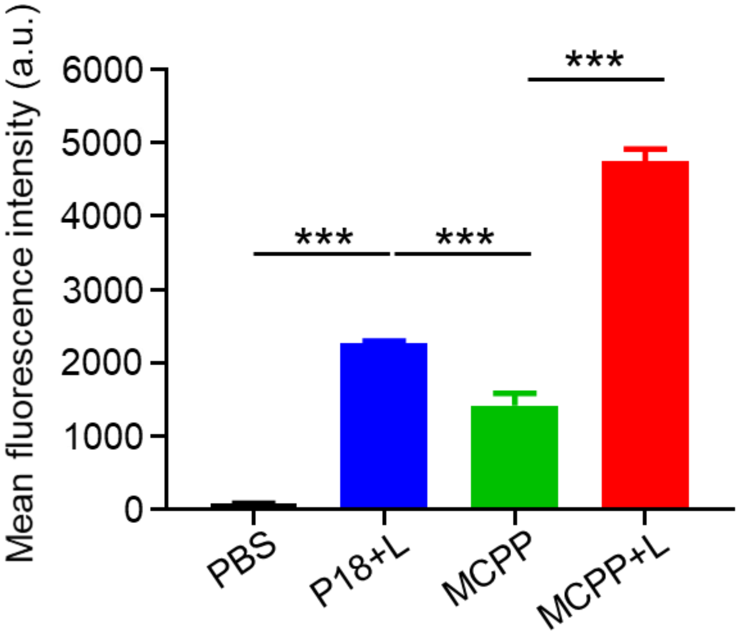

Figure S21. Quantitative analysis of ROS in CT26 cells after treated with PBS P18+L, MCPP, MCPP+L at a P18 dose of 5 μg/mL for 6 h, ROS was detected by DCFH-DA (green signal) (n = 3). (*$p < 0.05$; **$p < 0.01$; ***$p < 0.001$; ****$p < 0.0001$)

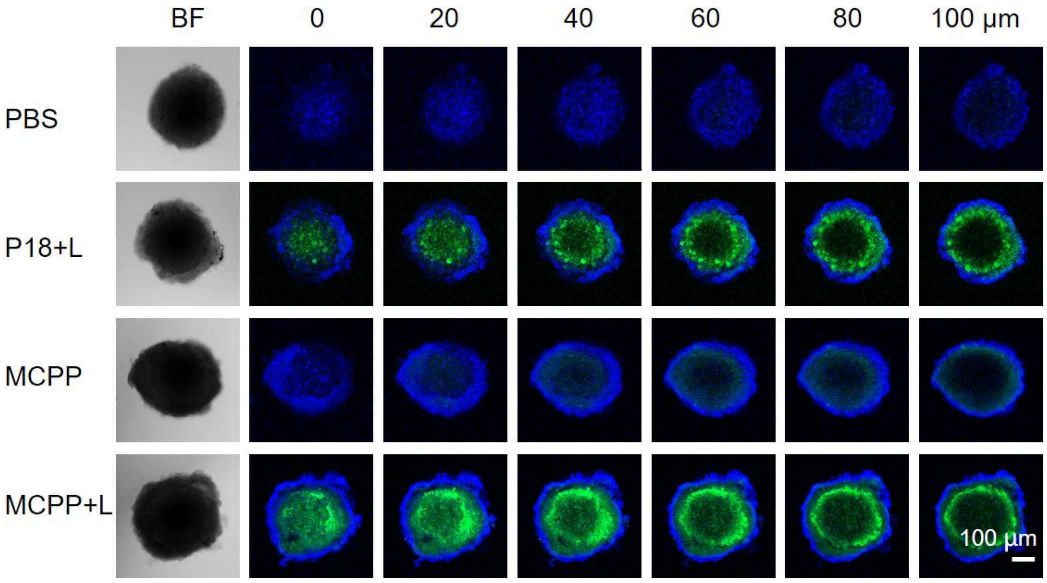

Figure S22. ROS detection in CT26 MCSs at different Z-axis after treated with PBS, P18+L, MCPP, MCPP+L at a P18 dose of 5 µg/mL for 6 h, ROS was detected by DCFH-DA (green signal). Scale bar = 100 µm.

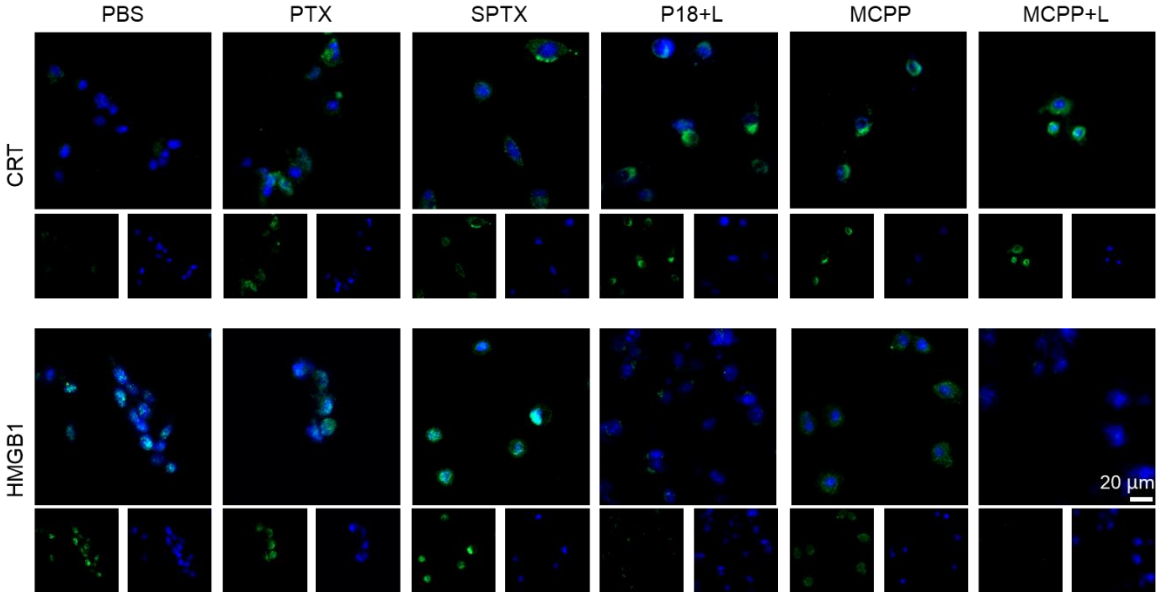

Figure S23. CRT and HMGB1 detection in CT26 cells after treated with PBS, PTX, SPTX, P18+L, MCPP, MCPP+L at a PTX dose of 30 µg/mL or P18 dose of 2.4 µg/mL for 12 h. Cells incubated with anti-CRT or anti-HMGB1 antibody and FITC- conjugated secondary antibody. After that, cells were counterstain with DAPI. CRT and HMGB1 fluorescence were detected by CLSM. Scale bar = 20 µm.

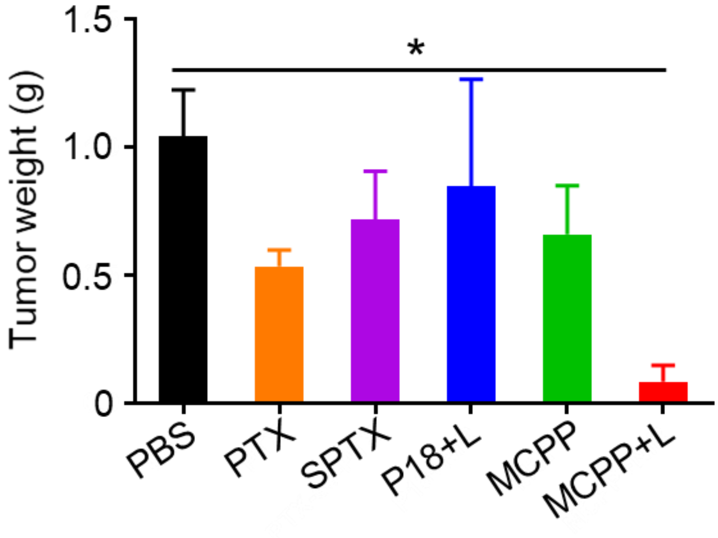

Figure S24. Tumor weight evaluation among different groups after treatment (n = 6). (*p < 0.05; **p < 0.01; ***p < 0.001; ****p < 0.0001)

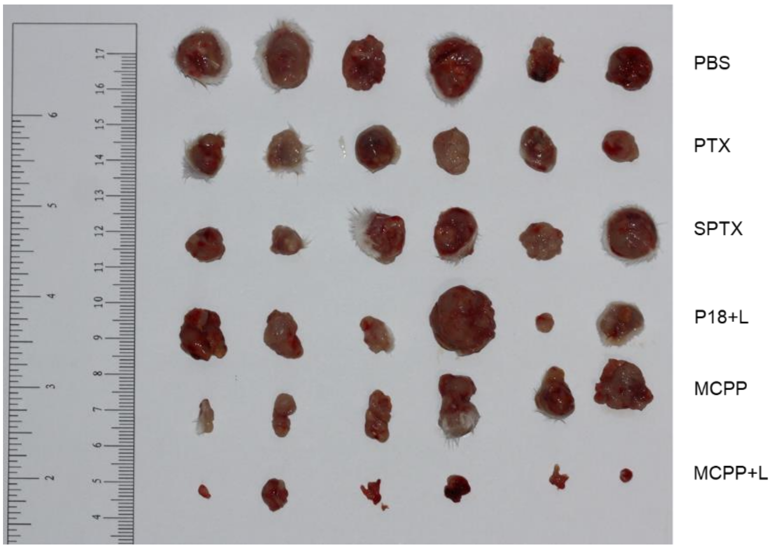

Figure S25. Photograph about the tumor from each group.

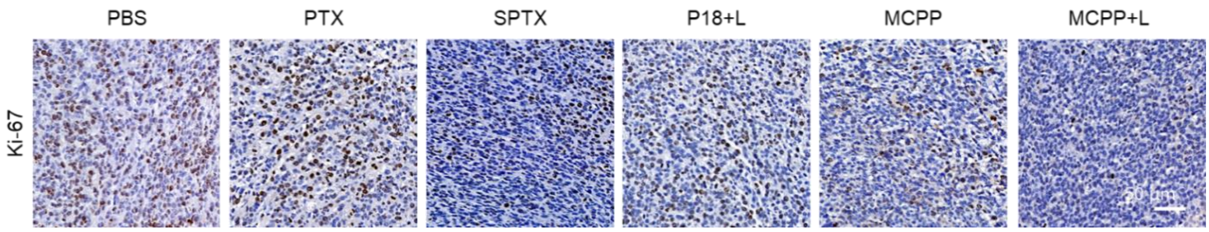

Figure S26. Representative figure of Ki-67 staining on tumor slide. Scale bar = 50 μm.

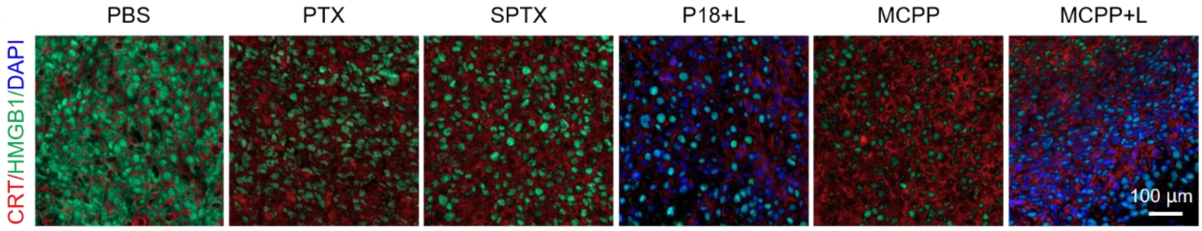

Figure S27. Representative images of CRT/HMGB1 multiplex immunohistochemistry staining on tumor slide. Scale bar = 100 μm.

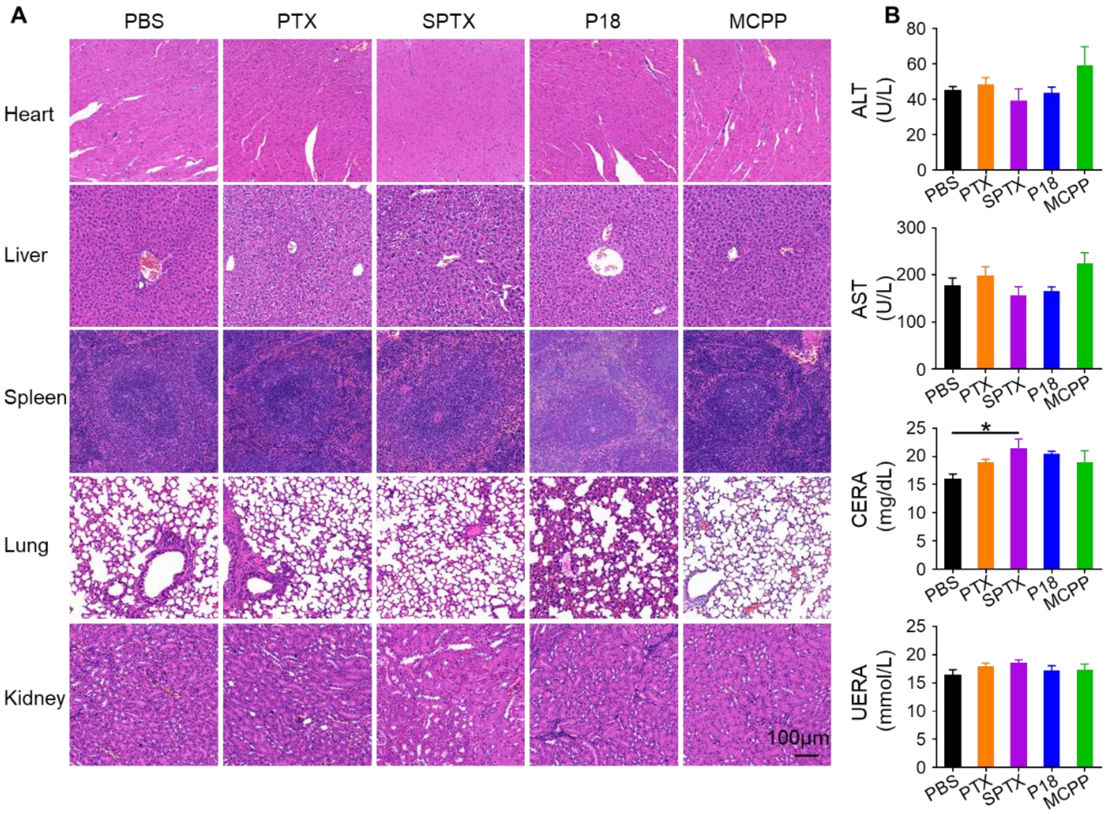

Figure S28. In vivo biosafety evaluation of different treatment. A. H&E staining of major organ from PBS, PTX, SPTX, P18, MCPP groups. B. Blood biochemical tests of mice from PBS, PTX, SPTX, P18, MCPP groups. Alanine aminotransferase (ALT), aspartate

aminotransferase (AST), creatinine (CERA), Urea (UREA) (n = 5). (*$p < 0.05$; **$p < 0.01$; ***$p < 0.001$; ****$p < 0.0001$)

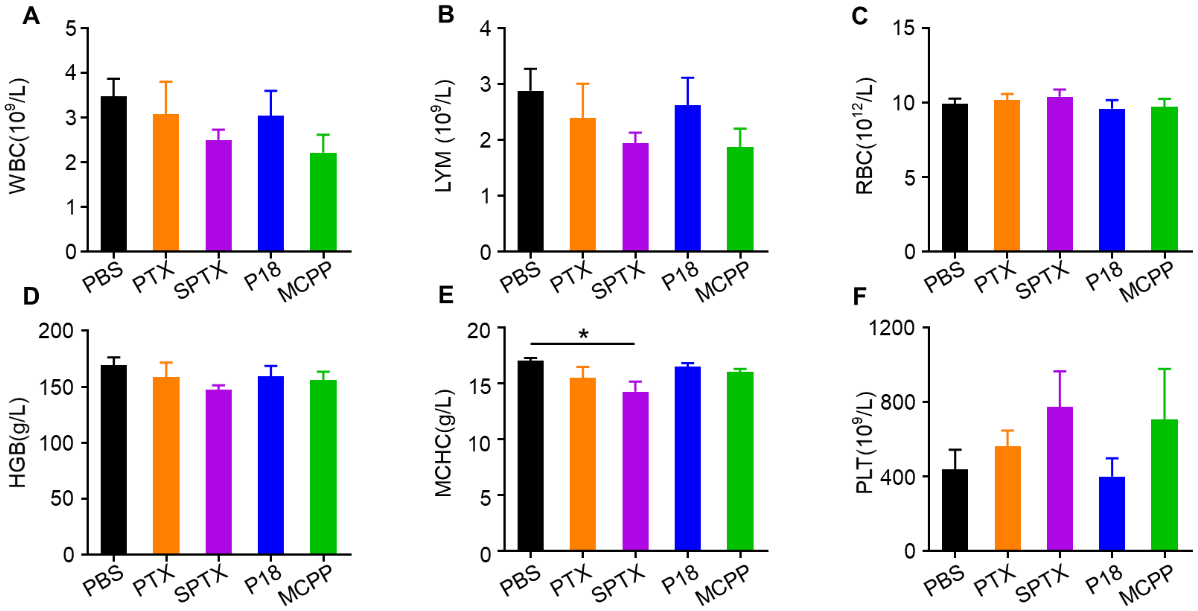

Figure S29. Routine blood tests of mice from PBS, PTX, SPTX, P18, MCPP groups. White blood cell count (WBC), lymphocyte ratio (LYM), red blood cell count (RBC), Hemoglobin concentration (HGB), Mean corpuscular hemoglobin content (MCH), blood platelet counts (PTL) (n = 5). (*$p < 0.05$; **$p < 0.01$; ***$p < 0.001$; ****$p < 0.0001$)

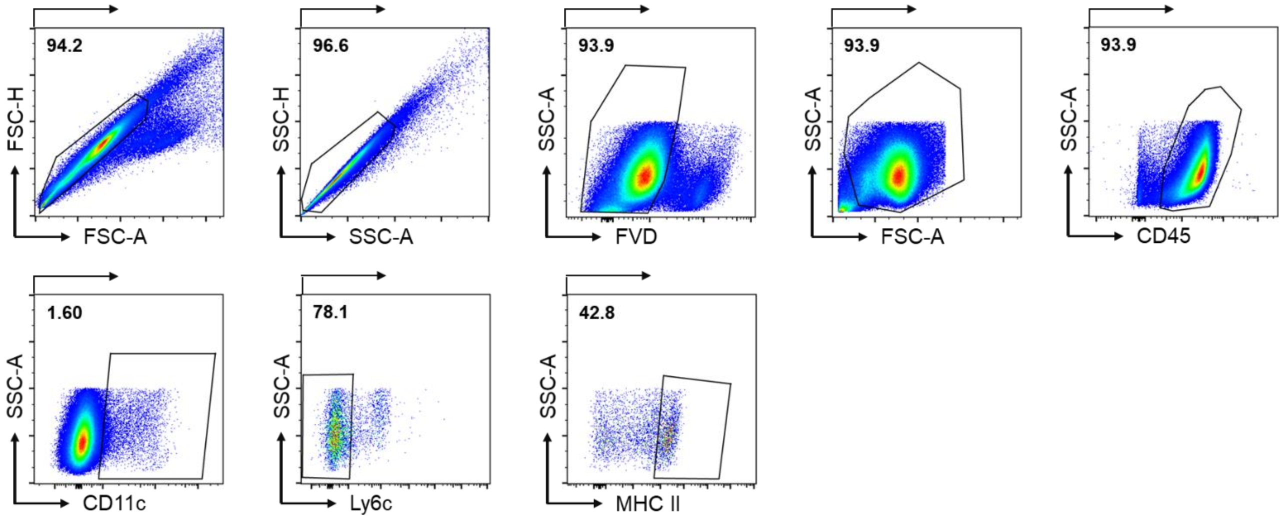

Figure S30. Dendritic cell gating strategy in flow cytometry analysis.

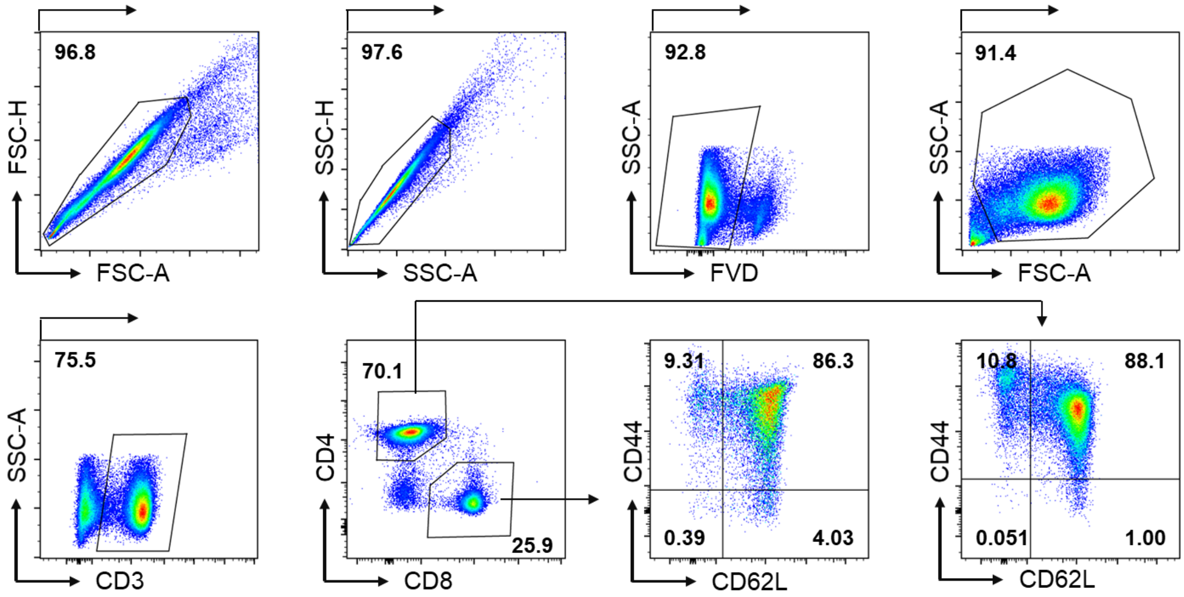

Figure S31. T cell gating strategy in flow cytometry analysis.

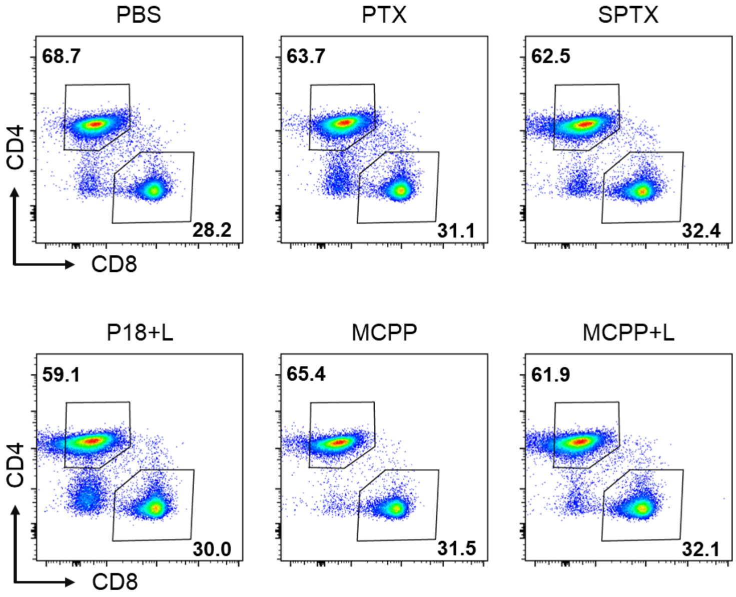

Figure S32. $CD4^+$ T and $CD8^+$ T cell proportion among different groups.

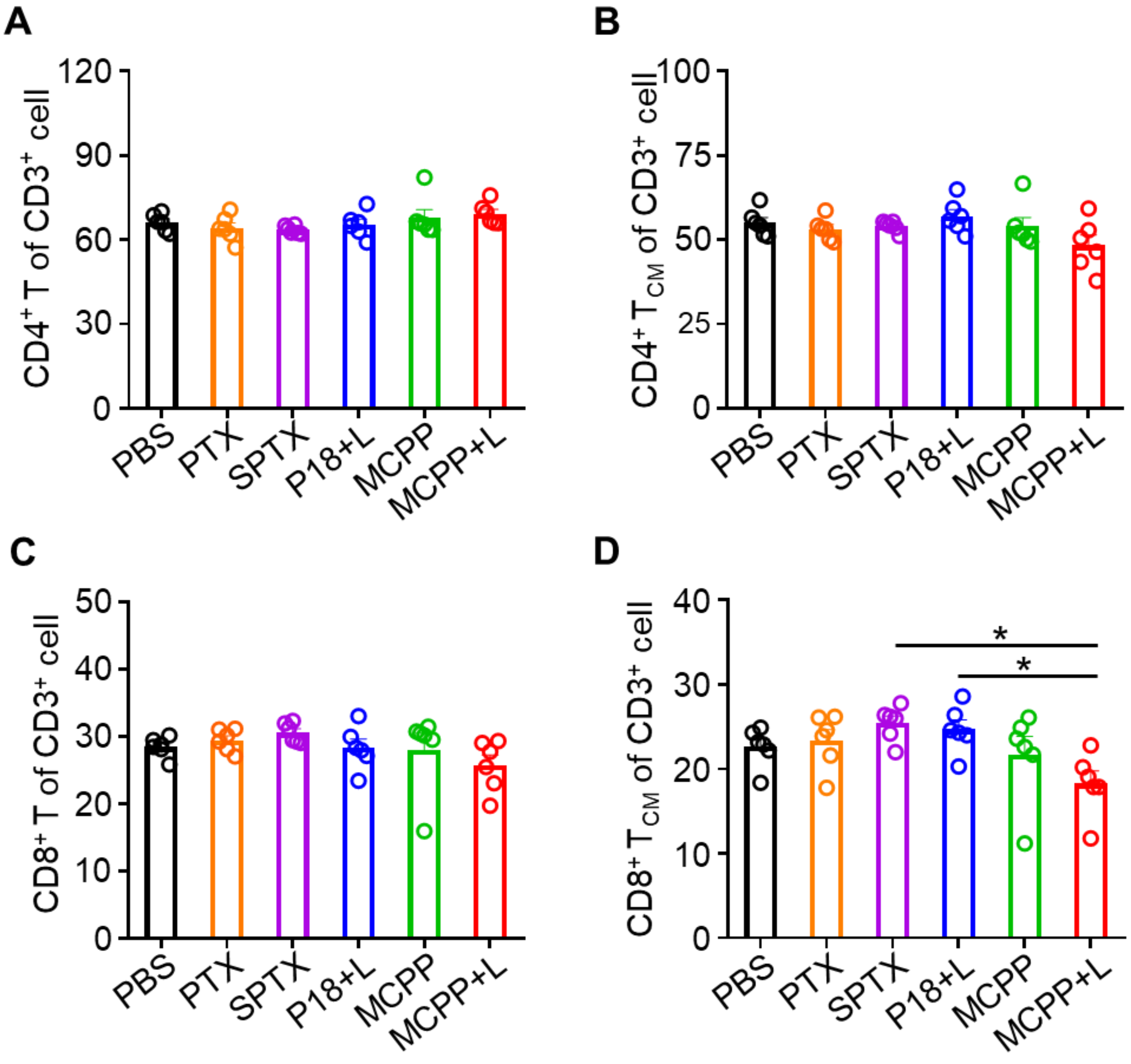

Figure S33. Quantitative analysis of $CD4^+$ T cell, central memory $CD4^+$ T cell, $CD8^+$ T cell, central memory $CD8^+$ T cell (n = 6). (*$p < 0.05$; **$p < 0.01$; ***$p < 0.001$; ****$p < 0.0001$)
